# Simultaneous activity and attenuation estimation in TOF-PET with TV-constrained nonconvex optimization

**Published:** 2023-03-29

**Authors:** Zhimei Ren, Emil Y. Sidky, Rina Foygel Barber, Chien-Min Kao, Xiaochuan Pan

**Affiliations:** Dept. of Statistics, University of Chicago; Dept. of Radiology, University of Chicago; Dept. of Statistics, University of Chicago; Dept. of Radiology, University of Chicago; Dept. of Radiology, University of Chicago

**Keywords:** Image reconstruction in TOF-PET, simultaneous activity/attenuation estimation, large-scale nonconvex optimization, alternating direction method of multipliers

## Abstract

An alternating direction method of multipliers (ADMM) framework is developed for nonsmooth biconvex optimization for inverse problems in imaging. In particular, the simultaneous estimation of activity and attenuation (SAA) problem in time-of-flight positron emission tomography (TOF-PET) has such a structure when maximum likelihood estimation (MLE) is employed. The ADMM framework is applied to MLE for SAA in TOF-PET, resulting in the ADMM-SAA algorithm. This algorithm is extended by imposing total variation (TV) constraints on both the activity and attenuation map, resulting in the ADMM-TVSAA algorithm. The performance of these algorithms is illustrated using the standard maximum likelihood activity and attenuation estimation (MLAA) algorithm as a reference.

## I. Introduction

Nuclear medicine imaging modalities such as single-photon emission computed tomography (SPECT) and positron emission tomography (PET) require the input of a gamma ray attenuation map for quantitatively accurate imaging. The combination of nuclear medicine imaging with other image modalities such as X-ray computed tomography (CT) [1], [2] or magnetic resonance imaging (MRI) [3] provides a means for estimating the necessary attenuation map. There are, however, challenges in the separate attenuation map estimation. Use of CT-based attenuation maps requires extrapolation of the photon attenuation map from the diagnostic X-ray energy range to 511 keV and registration of the PET and CT imaging, which can be particularly difficult in the presence of motion [4]. The use of MRI to estimate a synthetic CT image is further complicated by the fact that bone and air have similar gray values in MRI while bone has a significantly higher attenuation coefficient for gamma rays.

To avoid a separate measurement for obtaining the gamma ray attenuation map, a long-standing inverse problem of interest has been to simultaneously estimate the attenuation and activity distributions from emission data alone [5], [6]. To address simultaneous activity and attenuation (SAA) estimation, Nuyts *et al.* [6] use maximum likelihood to invert the algebraic SAA model, and they find that accurate activity distributions can be recovered by appropriately regularizing the attenuation map. The regularization involves the use of Gibbs and intensity priors on the attenuation distribution that encourage local smoothness and clustering of values around known attenuation values for tissues in the scanned subject. Another interesting result for the SAA problem is obtained in considering time-of-flight positron emission tomography (TOF-PET) [7]. Defrise *et al.* [8] exploit an analytic range condition [9], [10] for the continuous TOF-PET model and obtain a uniqueness result that the attenuation factor and activity can be determined up to a multiplicative constant. Returning to the SAA algebraic model for TOF-PET, a comprehensive study of this inverse problem using maximum likelihood estimation is presented in Rezaei *et al.* [11], where it is found that the activity and attenuation maps can be recovered if the timing resolution of the TOF measurements is sufficiently high and if support constraints are exploited. We note an intriguing extension of the SAA problem where the background radiation from Lutetium-176, present in PET scintillators composed of either lutetium oxyorthosilicate (LSO) or lutetium-yttrium orthosilicate (LYSO), is exploited to provide additional information on the subject’s attenuation map without the need for a separate scan [12].

In this work, we seek to build off of Ref. [11] and develop an image reconstruction framework for the SAA problem in TOF-PET that can incorporate nonsmooth, convex constraints in the maximum likelihood estimation. Such constraints can help to stabilize the image reconstruction when noise is present and can possibly extend the range of activity and attenuation factor recovery, possibly relaxing the requirements on the coincidence timing resolution and knowledge of the activity and attenuation distribution support. Of particular interest, here, is the use of total variation (TV) constraints on both activity and attenuation distributions. We have previously exploited such constraints in the context of nuclear medicine imaging; in Refs. [13] and [14] TV constraints are exploited to enable sparse-data sampling configurations in SPECT and PET, respectively. In Ref. [15], a similar methodology is used for image reconstruction in low-count list-mode TOF-PET.

The image reconstruction algorithms developed in Refs. [13]–[15] are all instances of a general primal-dual (PD) solver for nonsmooth convex optimization developed by Chambolle and Pock [16], [17]. The optimization problem posed by applying TV-constraints to the SAA estimation problem, however, is nonsmooth and nonconvex. In our recent work, we develop a framework for such problems in imaging, where the optimization can be split into convex terms plus differentiable terms that are possibly nonconvex [18]. This framework is based on the alternating direction method of multipliers (ADMM) [19] in a way that is closely related to the PD algorithm. This framework has been successfully applied to the nonsmooth and nonconvex optimization problem that arises in spectral computed tomography (CT) when the spectral response of the measurement is included in the data model [20]. Here, we modify this framework to address biconvex optimization and apply it to the SAA estimation problem with convex constraints. The SAA data model and imaging problem are specified in Sec. II, where we then develop an ADMM algorithm to solve the associated optimization problem. As the focus of this work is mainly on the SAA inverse problem, we conduct a number of studies on noiseless TOF-PET data in Sec. III that explore the range of TOF-PET parameters that allow exact recovery of activity and attenuation factors. Also presented in this section are results with noisy data that demonstrate the stability of the proposed algorithm. In Sec. IV the results are discussed and the conclusions of the work are given.

## II. Image reconstruction model and algorithms

In presenting the SAA algorithm TOF-PET, we consider a two dimensional (2D) simulation where the lines-of-response (LORs) are organized in parallel-ray fashion and are specified in the same way that the 2D Radon transform is parameterized. For the TOF-PET model, the Radon transform is modified by including weighted line-integration that accounts for TOF information that helps to localize the positron-electron annihilation along a given LOR. After specifying the TOF-PET data model, the MLAA algorithm from Rezaei *et al.* [11] is briefly summarized. We then present the nonconvex ADMM algorithm that performs SAA estimation with nonsmooth convex constraints.

### A. TOF-PET modelling

The measurement model for the mean data in TOF-PET is

(1) $$c_{i\ell }=\exp\left[-P_{\ell }^{⊤}\mu \right]\cdot T_{i\ell }^{⊤}\lambda$$

where λ and μ are the unknown activity and attenuation maps, respectively; $T_{i\ell k}$ is the TOF sensitivity matrix element for TOF window i, LOR ℓ, and image pixel k; $P_{\ell k}$ is the X-ray projection matrix element for LOR ℓ and pixel k. For defining the TOF projection matrix T, the TOF window sensitivity along the LOR is specified as

$$w_{i}(t)=\exp\left[-{\left(t-t_{i}\right)}^{2}/\left(2\sigma_{\text{TOF}}\right)\right],$$

where the sampling along the LOR is half of the full-width-half-maximum (FWHM) of this Gaussian distribution

$$\Delta t=t_{i+1}-t_{i}=\text{FWHM}/2=\sqrt{2\log2}\cdot \sigma_{\text{TOF}}.$$

For this work, scatter coincidences and random events are not considered.

### B. Imaging model based on nonconvex optimization

We consider performing SAA using likelihood maximization, where the measured coincidence count data are assummed to follow a multivariate Poisson distribution

$$C_{i\ell }\sim \mathrm{Poisson}\left(c_{i\ell }\right).$$

, Equivalently, this estimation is performed by minimization if the negative log-likelihood,

(2) $$\begin{array}{l} l(\lambda ,\mu )=\sum_{i\ell }\left\{c_{i\ell }-C_{i\ell }\cdot \log c_{i\ell }\right\}=\\ \sum_{i\ell }\left\{\exp\left(-P_{\ell }^{⊤}\mu \right)\cdot T_{i\ell }^{⊤}\lambda -C_{i\ell }\cdot \left(-P_{\ell }^{⊤}\mu +\log\left(T_{i\ell }^{⊤}\lambda \right)\right)\right\}. \end{array}$$

The optimization problem of interest is

(3) $$\lambda ,\mu =\underset{\lambda ,\mu }{\arg\min}\left\{l(\lambda ,\mu )|1^{⊤}\lambda =N_{\text{total}\;}\right\},$$

where l is the negative log-likelihood in Eq. (2); **1** is vector of size λ with unit entries so that $1^{⊤}\lambda$ is equivalent to summation over λ; and $N_{\text{total}}$ is the total number of annihilations. The constraint on the total number of annihilations is used to overcome the constant ambiguity in the SAA estimation problem [8]. This constraint is enforced in this work instead of the object support constraint investigated in Rezaei *et al.* [11].

### C. Summary of MLAA

To solve this imaging model, Rezaei *et al.* [11] developed the MLAA algorithm. For completeness, we write the MLAA update steps including a minor modification in Eq. (6) that accomodates the constraint on the total number of annihilations:

(4) $$a_{\ell }=\exp\left[-\sum_{k}P_{\ell k}\mu_{k}\right]\;\;\forall \ell ,$$

(5) $$\lambda_{k}\leftarrow \frac{\lambda_{k}}{\sum_{i\ell }a_{\ell }T_{i\ell k}}\sum_{i\ell }\left\{T_{i\ell k}\left(\frac{C_{i\ell }}{\sum_{k^{\prime }}T_{i\ell k^{\prime }}\lambda_{k^{\prime }}}\right)\right\}\;\;\;\;\forall k,$$

(6) $$\lambda \leftarrow \lambda \left(\frac{N_{\text{total}}}{\sum_{k}\lambda_{k}}\right),$$

(7) $$\mu_{k}\leftarrow \mu_{k}+\frac{\sum_{i\ell k^{\prime }}P_{\ell k}\left(a_{\ell }T_{i\ell k^{\prime }}\lambda_{k^{\prime }}\;-\;C_{i\ell }\right)}{\sum_{i\ell k^{\prime }}P_{\ell k^{\prime }}P_{\ell k}a_{\ell }\sum_{k^{\prime \prime }}T_{i\ell k^{\prime \prime }}\lambda_{k^{\prime \prime }}}\;\;\;\;\forall k.$$

The MLAA algorithm essentially alternates between updating λ with a Poisson likelihood EM step and μ with a Poisson transmission likelihood optimization step. In this MLAA implementation the extra update step in Eq. (6) enforces the constraint on the total number of annihilations. For MLAA the activity λ should have a strictly positive initialization, and this quantity will remain non-negative during the iteration. The attenuation map μ can be negative unless a non-negativity constraint is included.

Early stopping of the iteration is the primary means of performing regularization with MLAA, but explicit regularization can also be included with the use of Gibbs smoothing [21], [22]. In this work, we develop a framework for SAA which can include nonsmooth regularization.

### D. ADMM for nonsmooth and biconvex optimization

The general convex optimization problem that ADMM solves takes the form

$$\underset{x,y}{\min}\{f(x)+g(y)|Ax+By=c\},$$

where f and g are convex and possibly non-smooth functions; A and B are linear operators; x, y and c are vectors. The steps of the ADMM algorithm are

(8) $$\begin{array}{l} x\leftarrow \underset{x^{\prime }}{\arg\min}\{f\left(x^{\prime }\right)+u^{⊤}Ax^{\prime }\\ +\frac{1}{2}{\left\|Ax^{\prime }+By-c\right\|}_{\Sigma }^{2}+\frac{1}{2}{\left\|x^{\prime }-x\right\|}_{H_{f}}^{2}\} \end{array}$$

(9) $$\begin{array}{l} y\leftarrow \underset{y^{\prime }}{\arg\min}\{g\left(y^{\prime }\right)+u^{⊤}By^{\prime }\\ +\frac{1}{2}{\left\|Ax+By^{\prime }-c\right\|}_{\Sigma }^{2}+\frac{1}{2}{\left\|y^{\prime }-y\right\|}_{H_{g}}^{2}\} \end{array}$$

(10) u←u+Σ(Ax+By−c),

where Σ, $H_{f}$, and $H_{g}$ are symmetric positive definite, and $\|v{\|}_{M}^{2}\equiv v^{⊤}Mv$ for any symmetric positive definite matrix M. Because optimizing the TOF-PET likelihood is a non-convex optimization, the ADMM algorithm does not directly apply. The TOF-PET likelihood function, however, is biconvex; i.e. fixing either λ or μ, the likelihood is a convex function in the other variable.

The ADMM algorithm can be modified to accommodate a biconvex function, and we consider the case that g is a biconvex function

$$g(y)=g\left(y_{1},y_{2}\right),$$

where y is the concatenation of $y_{1}$ and $y_{2}$; and $g\left(y_{1},\cdot \right)$ and $g\left(\cdot ,y_{2}\right)$ are convex functions for fixed $y_{1}$ and $y_{2}$, respectively. To accommodate the biconvexity of g, the second update equation, Eq. (9), is replaced by an inner iteration with the following update equations

(11) $$\begin{array}{l} y_{1}\leftarrow \underset{y_{1}^{\prime }}{\arg\min}\{g\left(y_{1}^{\prime },y_{2}\right)+u^{⊤}B\left(y_{1}^{\prime },y_{2}\right)\\ +\frac{1}{2}{\left\|Ax+B\left(y_{1}^{\prime },y_{2}\right)-c\right\|}_{\Sigma }^{2}+\frac{1}{2}{\left\|\left(y_{1}^{\prime },y_{2}\right)-\left(y_{1},y_{2}\right)\right\|}_{H_{g}}^{2}\} \end{array}$$

(12) $$\begin{array}{l} y_{2}\leftarrow \underset{y_{2}^{\prime }}{\arg\min}\{g\left(y_{1},y_{2}^{\prime }\right)+u^{⊤}B\left(y_{1},y_{2}^{\prime }\right)\\ +\frac{1}{2}{\left\|Ax+B\left(y_{1},y_{2}^{\prime }\right)-c\right\|}_{\Sigma }^{2}+\frac{1}{2}{\left\|\left(y_{1},y_{2}^{\prime }\right)-\left(y_{1},y_{2}\right)\right\|}_{H_{g}}^{2}\}. \end{array}$$

The inner loop consists of alternating between Eqs. (11) and (12) for a predetermined number of iterations $N_{y}$, where $N_{y}\geq 1$. After the inner loop is completed, the ADMM iteration continues with Eq. (10) after the following assignment

$$y=\left(y_{1},y_{2}\right).$$

This inner loop, specified in Eqs. (11) and (12), is computationally efficient if multiplication by the matrix B is efficient; this is the case in our application because we consider B=I where I is the identity matrix. Note that multiplication by A is not performed within this inner iteration because the matrix A only appears in the term Ax which is computed before entering the inner loop.

### E. ADMM for large-scale tomographic image reconstruction

For the large-scale optimization problems that arise in tomographic image reconstruction, the update step in Eq. (8) can be problematic because of the term Ax, which appears in the minimization over x. The matrix A usually contains the system matrix for the imaging model, and computation of Ax can be expensive particularly for 3D imaging; thus numerical solution of Eq. (8) may not be feasible. This “expensive inner loop” problem can be circumvented by linearization, i.e. by including the additional term $\frac{1}{2}{\left\|x^{\prime }-x\right\|}_{H_{f}}^{2}$ in Eq. (8) [18], [23], resulting in an algorithm closely related to the primal-dual (PD) algorithm of Chambolle and Pock [16], [17]. Considering only scalar step-size parameters, i.e.

Σ=σI,

the metric $H_{f}$ in Eq. (8) is set to

(13) $$H_{f}=I/\tau -\sigma A^{⊤}A.$$

This choice cancels the Ax term in Eq. (2), and the requirement that $H_{f}$ be positive definite yields a constraint on the step-sizes σ and τ. In the context of the image reconstruction problem, we also have

$$H_{g}=0;\;\;\;\;B=-I;\;\;\;\;c=0.$$

The ADMM generic optimization problem becomes

(14) $$\underset{x,y}{\min}\{f(x)+g(y)\}|Ax-y=0,$$

and the algorithm for convex optimization is then specified by the following update equations

(15) $$x\leftarrow \underset{x^{\prime }}{\arg\min}\{f\left(x^{\prime }\right)+x^{\prime ⊤}A^{⊤}(u+\sigma (Ax-y))+\frac{1}{2\tau }{\left\|x^{\prime }-x\right\|}^{2}\}$$

(16) $$y\leftarrow \underset{y^{\prime }}{\arg\min}\left\{g\left(y^{\prime }\right)-u^{⊤}y^{\prime }+\frac{\sigma }{2}{\left\|Ax-y^{\prime }\right\|}^{2}\right\}$$

(17) u←u+σ(Ax−y).

Aside from minor details, this set of update equations is equivalent to the PD algorithm, but as a starting point to modify the update steps for non-convex optimization, this form is more convenient because both f and g functions appear directly in the updates. In contrast, the PD algorithm dualizes g and the convex conjugate $g^{⋆}$ is needed. If it is desired to apply PD to non-convex g, figuring out what to put in place of $g^{⋆}$, while possible [24], adds another layer of complication to the algorithm development.

The modification of the linearized ADMM updates for addressing the case where g is biconvex replaces Eq. (16) with inner loop update equations

(18) $$y_{1}=\underset{y_{1}^{\prime }}{\arg\min}\{g\left(y_{1}^{\prime },y_{2}\right)-u^{⊤}\left(y_{1}^{\prime },y_{2}\right)\;+\frac{\sigma }{2}{\left\|Ax-\left(y_{1}^{\prime },y_{2}\right)\right\|}^{2}\}$$

(19) $$\begin{array}{l} y_{2}=\underset{y_{2}^{\prime }}{\arg\min}\{g\left(y_{1},y_{2}^{\prime }\right)-u^{⊤}\left(y_{1},y_{2}^{\prime }\right)\\ \;\;\;\;\;+\frac{\sigma }{2}{\left\|Ax-\left(y_{1},y_{2}^{\prime }\right)\right\|}^{2}\}. \end{array}$$

Convergence of this modified ADMM algorithm for biconvex functions is not theoretically guaranteed and thus convergence is demonstrated empirically.

### F. ADMM for SAA in TOF-PET

The instantiation of ADMM for SAA estimation by minimization of the negative log-likelihood is covered here in detail. The optimization problem of interest is

(20) $$\lambda ,\mu =\underset{\lambda ,\mu }{\arg\min}\left\{l(\lambda ,\mu )|1^{⊤}\lambda =N_{\text{total}\;},\;\;\;\;P\mu \geq 0\right\},$$

which is essentially the same as the optimization problem in Eq. (3). The only difference is that an additional non-negativity constraint is introduced on the sinogram of the attenuation map.

To map the optimization problem in Eq. (20) onto the generic ADMM optimization in Eq. (14), the primal, splitting, and dual variables x, y, and u, are respectively assigned as

$$x=\left(\begin{array}{c} \lambda \\ \mu \end{array}\right),y=\left(\begin{array}{l} y_{\lambda }\\ y_{\mu } \end{array}\right),u=\left(\begin{array}{l} u_{\lambda }\\ u_{\mu } \end{array}\right).$$

The linear system A is assigned as

$$A=\left(\begin{array}{ll} T & 0\\ 0 & P \end{array}\right).$$

The convex function f is used to represent the constraint on the total number of annihilations by setting

(21) $$f(\lambda ,\mu )=\delta \left(1^{⊤}\lambda =N_{\text{total}\;}\right),$$

where δ is the convex indicator function, which is zero if the conditional argument is true and infinity otherwise. The biconvex function g accounts for the remaining terms in Eq. (20)

(22) $$\begin{array}{l} g\left(y_{\lambda },y_{\mu }\right)=L\left(y_{\lambda },y_{\mu }\right)+\delta \left(y_{\mu }\geq 0\right),\\ L\left(y_{\lambda },y_{\mu }\right)=\sum_{i\ell }\{\exp\left(-y_{\mu ,\;\ell }\right)\cdot y_{\lambda ,\;i\ell }\\ \;\;\;\;\;\;\;\;\;\;\;\;\;\;\;\;\;\;\;\;-C_{i\ell }\cdot \left(-y_{\mu ,\;\ell }+\log\left(y_{\lambda ,\;i\ell }\right)\right)\}, \end{array}$$

where

l(λ,μ)=L(Tλ,Pμ).

#### Parametrization of the step-sizes:

Step-size selection is a critical issue for first-order, large-scale optimization algorithms. There can be much flexibility in the step-size selection, and it is important to select a minimal set of free parameters that are effective for algorithm efficiency but not too cumbersome in the tuning procedure. Because the system matrix A for SAA is block-diagonal, a slight generalization of the ADMM linearization is considered. The metric $H_{f}$ is written as

$$H_{f}=\left(\begin{array}{cc} H_{\lambda } & 0\\ 0 & H_{\mu } \end{array}\right),$$

$$H_{\lambda }=\frac{I}{\tau_{\lambda }}-\sigma_{\lambda }T^{⊤}T,$$

$$H_{\mu }=\frac{I}{\tau_{\mu }}-\sigma_{\mu }P^{⊤}P,$$

and the step-size parameters are chosen according to

$$\sigma_{\lambda }\tau_{\lambda }=1/\|T{\|}_{2}^{2},\;\;\;\;\sigma_{\mu }\tau_{\mu }=1/\|P{\|}_{2}^{2},$$

where ${\left\|M\right\|}_{2}$ is the largest singular value of the matrix M. With four step-size parameters and two equality constraints, there are two free step-size parameters. Specifically, the step-size ratios, $\rho_{\lambda }$ and $\rho_{\mu }$, are chosen to be the free parameters that need to be tuned:

(23) $$\sigma_{\lambda }=\rho_{\lambda }/\|T{\|}_{2},\tau_{\lambda }=1/\left(\rho_{\lambda }\|T{\|}_{2}\right),$$

(24) $$\sigma_{\mu }=\rho_{\mu }/\|P{\|}_{2},\tau_{\mu }=1/\left(\rho_{\mu }\|P{\|}_{2}\right).$$

Tuning of $\rho_{\lambda }$ and $\rho_{\mu }$ is a necessary step any time the T or P matrices are changed due to, for example, a change in scan configuration or sampling pattern.

#### *The* x-*update:*

For the SAA problem in TOF-PET the x-update in Eq. (15) splits into two optimization problems

(25) $$\begin{array}{l} \lambda \leftarrow \underset{\lambda^{\prime }}{\arg\min}\{\lambda^{\prime ⊤}T^{⊤}\left(u_{\lambda }+\sigma_{\lambda }\left(T\lambda -y_{\lambda }\right)\right)\\ +\frac{1}{2\tau_{\lambda }}{\left\|\lambda^{\prime }-\lambda \right\|}^{2}|1^{⊤}\lambda^{\prime }=N_{\text{total}\;}\}, \end{array}$$

(26) $$\mu \leftarrow \underset{\mu^{\prime }}{\arg\min}\{\mu^{\prime ⊤}P^{⊤}\left(u_{\mu }+\sigma_{\mu }\left(P\mu -y_{\mu }\right)\right)$$

(27) $$+\frac{1}{2\tau_{\mu }}{\left\|\mu^{\prime }-\mu \right\|}^{2}\},$$

where the convex function f from Eq. (21) is incorporated in the λ-update equation. The optimization problem for the μ-update in Eq. (26) is solved by setting the gradient of the objective function to zero and solving for $\mu^{\prime }$

(28) $$\begin{array}{l} \mu \leftarrow \mu -\tau_{\mu }P^{⊤}{\bar{u}}_{\mu },\\ {\bar{u}}_{\mu }=u_{\mu }+\sigma_{\mu }\left(P\mu -y_{\mu }\right). \end{array}$$

For the λ optimization problem in Eq. (25), the total annihilation count equality constraint is accounted for using the technique of Lagrange multipliers. The objective function is augmented introducing the scalar Lagrange multiplier ν, becoming

$$\begin{array}{l} \phi \left(\nu ,\lambda^{\prime }\right)=\nu \left(1^{⊤}\lambda^{\prime }-N_{\text{total}\;}\right)+\\ \;\lambda^{\prime ⊤}T^{⊤}\left(u_{\lambda }+\sigma_{\lambda }\left(T\lambda -y_{\lambda }\right)\right)+\frac{1}{2\tau_{\lambda }}{\left\|\lambda^{\prime }-\lambda \right\|}^{2}. \end{array}$$

The augmented objective function is minimized by taking its gradient, setting it to zero, and solving for ν and $\lambda^{\prime }$. The derivative with respect to ν gives back the total annihilation count constraint equation, and the gradient with respect to $\lambda^{\prime }$ is

$$\frac{\partial \phi \left(\nu ,\;\lambda^{\prime }\right)}{\partial \lambda^{\prime }}=\nu 1+T^{⊤}\left(u_{\lambda }+\sigma_{\lambda }\left(T\lambda -y_{\lambda }\right)\right)+\frac{1}{\tau_{\lambda }}\left(\lambda^{\prime }-\lambda \right)$$

Setting this gradient to zero yields

(29) $$\begin{array}{l} 0=\nu 1+T^{⊤}{\bar{u}}_{\lambda }+\frac{1}{\tau_{\lambda }}\left(\lambda^{\prime }-\lambda \right),\\ {\bar{u}}_{\lambda }=u_{\lambda }+\sigma_{\lambda }\left(T\lambda -y_{\lambda }\right). \end{array}$$

Both ν and $\lambda^{\prime }$ are unknown in Eq. (29), but this problem can be resolved by invoking the constraint equation $1^{⊤}\lambda^{\prime }=N_{\text{total}\;}$ by multiplying Eq. (29) through by **1**^Τ^:

$$0=\nu N_{\text{pix}\;}+1^{⊤}T^{⊤}{\bar{u}}_{\lambda }+\frac{1}{\tau_{\lambda }}\left(N_{\text{total}\;}-1^{⊤}\lambda \right),$$

and noting that $1^{⊤}1=N_{\text{pix}}$, where $N_{\text{pix}}$ is the total number of pixels in the activity map. Solving for ν and substituting back into Eq. (29) yields the λ-update

(30) $$\begin{array}{l} \lambda \leftarrow \lambda -\tau_{\lambda }\left(\nu 1+T^{⊤}{\bar{u}}_{\lambda }\right),\\ \nu =\frac{1}{\tau_{\lambda }N_{\text{pix}\;}}\left(1^{⊤}\lambda -N_{\text{total}\;}-\tau_{\lambda }1^{⊤}T^{⊤}{\bar{u}}_{\lambda }\right). \end{array}$$

Computationally, the updates in λ and μ are the most expensive steps in the ADMM algorithm because they involve forward- and back-projection of μ and ${\bar{u}}_{\mu }$, respectively, in addition to TOF forward- and back-projection of λ and ${\bar{u}}_{\lambda }$, respectively.

#### *The biconvex* y-*updates:*

The g function in Eq. (22) is biconvex in that it is convex in $y_{\lambda }$ if $y_{\mu }$ is fixed and *vice versa*. Splitting up the g function over the two update equations in Eqs. (18) and (19) yields

(31) $$\begin{array}{l} y_{\lambda }=\underset{y_{\lambda }^{\prime }}{\arg\min}\{\sum_{i\ell }\left(\exp\left(-y_{\mu ,\;\ell }\right)\cdot y_{\lambda ,\;i\ell }^{\prime }-C_{i\ell }\cdot \log\left(y_{\lambda ,\;i\ell }^{\prime }\right)\right)\\ \;-u_{\lambda }^{⊤}y_{\lambda }^{\prime }+\frac{\sigma_{\lambda }}{2}{\left\|y_{\lambda }^{\prime }-T\lambda \right\|}^{2}\}, \end{array}$$

and

(32) $$\begin{array}{l} y_{\mu }=\underset{y_{\mu }^{\prime }}{\arg\min}\{\sum_{i\ell }\left(\exp\left(-y_{\mu ,\;\ell }^{\prime }\right)\cdot y_{\lambda ,\;i\ell }+C_{i\ell }\cdot y_{\mu ,\;\ell }^{\prime }\right)\\ \;\;\;\;\;\;\;\;\;\;\;\;\;\;\;\;\;\;\;\;-u_{\mu }^{⊤}y_{\mu }^{\prime }+\frac{\sigma_{\mu }}{2}{\left\|y_{\mu }^{\prime }-P\mu \right\|}^{2}|y_{\mu }^{\prime }\geq 0\}, \end{array}$$

noting that the exp(${-y}_{\mu }$) · $y_{\lambda }$ term is the only one that mixes the $y_{\lambda }$ and $y_{\mu }$ variables and is therefore common to both minimization problems.

The minimization problems for the *y*-update are both separable over the components of $y_{\lambda }^{\prime }$ and $y_{\mu }^{\prime }$. The minimization over $y_{\lambda }^{\prime }$ in Eq. (31) is solved analytically by setting the gradient of the objective function to zero, yielding a quadratic equation. The resulting update equation is

(33) $$\begin{array}{l} y_{\lambda ,\;i\ell }=\left(b_{i\ell }+\sqrt{b_{i\ell }^{2}+4\sigma_{\lambda }C_{i\ell }}\right)/\left(2\sigma_{\lambda }\right)\\ b_{i\ell }=u_{\lambda ,\;i\ell }+\sigma_{\lambda }T_{i\ell }^{⊤}\lambda -\exp\left(-y_{\mu ,\;\ell }\right). \end{array}$$

In solving the quadratic equation the positive root is chosen because it results in physical non-negative values of $y_{\lambda ,\;i\ell }$.

Solving the minimization over $y_{\mu }^{\prime }$ in Eq. (32) is more involved because setting the gradient of the objective function to zero results in a transcendental equation, which requires the use of a numerical solver. The objective function is convex in $y_{\mu }^{\prime }$ and its derivatives are easily computed analytically. Thus Newton’s algorithm can be applied to obtain an efficient and accurate solution to Eq. (32). Both the first and second derivatives of the objective function are needed for Newton’s algorithm. Defining ψ to be the objective function of Eq. (32)

$$\begin{array}{l} \psi \left(y_{\mu }^{\prime }\right)=\sum_{i\ell }\left(\exp\left(-y_{\mu ,\;\ell }^{\prime }\right)\cdot y_{\lambda ,\;i\ell }+C_{i\ell }\cdot y_{\mu ,\;\ell }^{\prime }\right)\\ \;-u_{\mu }^{⊤}y_{\mu }^{\prime }+\frac{\sigma_{\mu }}{2}{\left\|y_{\mu }^{\prime }-P\mu \right\|}^{2}, \end{array}$$

the first derivative of ψ is

(34) $$\begin{array}{l} \frac{\partial \psi \left(y_{\mu }^{\prime }\right)}{\partial y_{\mu ,\;\ell }^{\prime }}=-\exp\left(-y_{\mu ,\;\ell }^{\prime }\right)\cdot y_{\lambda ,\;\ell }\\ \;\;\;\;\;\;\;\;\;\;\;\;\;\;\;\;\;\;\;+C_{\ell }-u_{\mu }+\sigma_{\mu }\left(y_{\mu ,\;\ell }^{\prime }-P_{\ell }^{⊤}\mu \right), \end{array}$$

where

$$y_{\lambda ,\;\ell }=\sum_{i}y_{\lambda ,\;i\ell },\;\;\;\;C_{\ell }=\sum_{i}C_{i\ell }.$$

The second derivative of ψ is

(35) $$\frac{\partial^{2}\psi \left(y_{\mu }^{\prime }\right)}{\partial y_{\mu ,\;\ell }^{\prime }}=\exp\left(-y_{\mu ,\;\ell }^{\prime }\right)\cdot y_{\lambda ,\;\ell }+\sigma_{\mu },$$

which is strictly positive. Thus Newton’s algorithm can be applied without any difficulties with the following update equation

(36) $$y_{\mu ,\;\ell }^{\prime }\leftarrow y_{\mu ,\;\ell }^{\prime }-\frac{\partial \psi \left(y_{\mu }^{\prime }\right)}{\partial y_{\mu ,\;\ell }^{\prime }}{\left(\frac{\partial^{2}\psi \left(y_{\mu }^{\prime }\right)}{\partial y_{\mu ,\;\ell }^{\prime }}\right)}^{-1}.$$

There is also the non-negativity constraint in Eq. (32), and this can be accounted for by thresholding negative values of $y_{\mu ,\;\ell }^{\prime }$ to zero after the Newton iteration is completed.

The proposed *y*-update involves two additional levels of iteration. The first additional level of iteration involves alternative between solving Eqs. (31) and (32). In the second additional level of iteration Eq. (32) is solved with the Newton iteration in Eq. (36). Nevertheless, these additional nested iterations do not negatively impact the efficiency of the overall algorithm because all of the iterations for the *y*-update separate over the components of y. The complete *y*-update computation takes less effort than computing, Tλ, the TOF data of an estimate of the activity map, λ. This is one of the useful aspects of the powerful splitting technique that ADMM exploits.

#### *The* u-*update:*

The final set of ADMM update equations involve updating the u variables. For SAA in TOF-PET, Eq. (17) becomes

(37) $$u_{\lambda }\leftarrow u_{\lambda }+\sigma_{\lambda }\left(T\lambda -y_{\lambda }\right),$$

(38) $$u_{\mu }\leftarrow u_{\mu }+\sigma_{\mu }\left(P\mu -y_{\mu }\right).$$

**Algorithm 1:** ADMM pseudocode for SAA estimation with biconvex optimization. Variables $\lambda ,\mu ,y_{\lambda },y_{\mu },{\overset{‾}{y}}_{\lambda },{\overset{‾}{y}}_{\mu },u_{\lambda }$, and $u_{\mu }$ are initialized to zero. Step size ratio parameters $\rho_{\lambda }$ and $\rho_{\mu }$ are chosen, and step size parameters $\sigma_{\lambda },\sigma_{\mu },\tau_{\lambda }$, and $\tau_{\mu }$ are determined according to Eqs. (23) and (24).

1:	**for** *k* ← 1, *N*_iter_ **do**
2:	$\bar{\lambda }=T^{⊤}\left(u_{\lambda }+\sigma_{\lambda }\left({\bar{y}}_{\lambda }-y_{\lambda }\right)\right)$
3:	$\nu =\frac{1}{\tau_{\lambda }N_{\text{pix}\;}}\left(1^{⊤}\lambda -N_{\text{total}}-\tau_{\lambda }1^{⊤}\bar{\lambda }\right)$
4:	$\lambda \leftarrow \lambda -\tau_{\lambda }(\nu 1+\bar{\lambda })$
5:	${\bar{y}}_{\lambda }=T\lambda$
6:	$\bar{\mu }=P^{⊤}\left(u_{\mu }+\sigma_{\mu }\left({\bar{y}}_{\mu }-y_{\mu }\right)\right)$
7:	$\mu \leftarrow \mu -\tau_{\mu }\bar{\mu }$
8:	${\bar{y}}_{\mu }=P\mu$
9:	$v_{\mu ,\ell }=\sum_{i}C_{i\ell }-\left(u_{\mu ,\;\ell }-\sigma_{\mu }{\bar{y}}_{\mu ,\;\ell }\right)\forall \ell$
10:	**for** *k*′ ← 1, $N_{y}$ **do** ▷ Biconvex alternation loop
11:	$b_{i\ell }=u_{\lambda ,\;i\ell }+\sigma_{\lambda }{\bar{y}}_{\lambda ,\;i\ell }-\exp\left(-y_{\mu ,\ell }\right)\forall i,\ell$
12:	$y_{\lambda ,\;i\ell }=\left(b_{i\ell }+\sqrt{b_{i\ell }^{2}+4\sigma_{\lambda }C_{i\ell }}\right)/\left(2\sigma_{\lambda }\right)\forall i,\;\ell$
13:	$y_{\lambda ,\ell }=\sum_{i}y_{\lambda ,\;i\ell }\forall \ell$
14:	$y_{\mu ,\ell }^{\prime }=0\;\;\;\;\forall \ell$ ▷ Initialize Newton iteration
15:	**for** *k*′′ ← 1, *N*_newt_ **do** ▷ Loop for solving Eq. (32)
16:	$\psi_{\ell }^{\left(1\right)}=-\exp\left(-y_{\mu ,\;\ell }^{\prime }\right)\cdot y_{\lambda ,\;\ell }+\sigma_{\mu }y_{\mu ,\;\ell }^{\prime }+v_{\mu ,\;\ell }$
17:	$\psi_{\ell }^{\left(2\right)}=-\exp\left(-y_{u}^{\prime }\right)\cdot y_{\lambda ,\;\ell }+\sigma_{\mu }\forall \ell$
18:	$y_{\mu ,\;\ell }^{\prime }\leftarrow y_{\mu ,\;\ell }^{\prime }-\psi_{\ell }^{\left(1\right)}\cdot {\left(\psi_{\ell }^{\left(2\right)}\right)}^{-1}\forall \ell$
19:	$y_{\mu ,\;\ell }^{\prime }\leftarrow \mathrm{pos}\left(y_{\mu ,\;\ell }^{\prime }\right)\forall \ell$ ▷ Nonneg., Eq. (32)
20:	**end for**
21:	$y_{\mu ,\;\ell }=y_{\mu ,\;\ell }^{\prime }\forall \ell$
22:	**end for**
23:	$u_{\lambda }\leftarrow u_{\lambda }+\sigma_{\lambda }\left({\bar{y}}_{\lambda }-y_{\lambda }\right)$
24:	$u_{\mu }\leftarrow u_{\mu }+\sigma_{\mu }\left({\bar{y}}_{\mu }-y_{\mu }\right)$
25:	**end for**

#### ADMM pseudocode for SAA estimation:

The *x*-, *y*-, and *u*-update equations are assembled into a complete pseudocode given in Algorithm 1. The expensive projection and back-projection computations are collected in as few lines as possible, and their results stored, to avoid unnecessary repetition of these burdensome operations. The first derivative computation from Eq. (34) is performed at lines 9 and 16, where line 9 collects all terms that are not dependent on $y_{\lambda }$ or $y_{\mu }^{\prime }$. The function pos(·) in line 19 returns the argument if it is nonnegative, otherwise it returns zero. For the results presented in this work, we only consider zero initialization for all of the algorithm variables. The choice of step-size ratios $\rho_{\lambda }$ and $\rho_{\mu }$ will impact the convergence rate of the algorithm, and these parameters must be tuned for optimal performance.

### G. ADMM for TV-constrained SAA in TOF-PET

The proposed ADMM framework for solving SAA estimation in TOF-PET allows for great flexibility in imposing convex constraints in the imaging optimization problem. Accordingly, we augment the total annihilation count and attenuation sinogram nonnegativity constraints in Eq. (20) with additional total variation constraints on the activity and attenuation maps

(39) $$\begin{array}{l} \lambda ,\mu =\underset{\lambda ,\mu }{\arg\min}\{l(\lambda ,\mu )|\|\lambda {\|}_{\text{TV}}\leq \gamma_{\lambda },\;\;\;\;\|\mu {\|}_{\text{TV}}\leq \gamma_{\mu },\\ \;1^{⊤}\lambda =N_{\text{total}\;},\;\;\;\;P\mu \geq 0,\} \end{array}$$

where $\|\cdot {\|}_{\text{TV}}$ is the isotropic TV seminorm; $\gamma_{\lambda }$ and $\gamma_{\mu }$ are the TV constraint values for the activity and attenuation maps, respectively. The additional TV constraints exploit gradient sparsity in in both the activity and attenuation that potentially improves accurate estimation of their corresponding images.

Because the novel aspect of this work is the treatment of the biconvex log-likelihood term, which is explained in detail in Sec. II-F, the ADMM instance for this optimization problem is covered in the Supplemental Document. The ADMM algorithm for TV-constrained SAA estimation (ADMM-TVSAA) is also designed so that it makes use of the same step size ratio parameters as discussed for Algorithm 1. Because of the additional constraints, the TV constraint values $\gamma_{\lambda }$ and $\gamma_{\mu }$ become additional parameters of the algorithm.

## III. Results with a 2D TOF-PET simulation

**Fig. 1.**
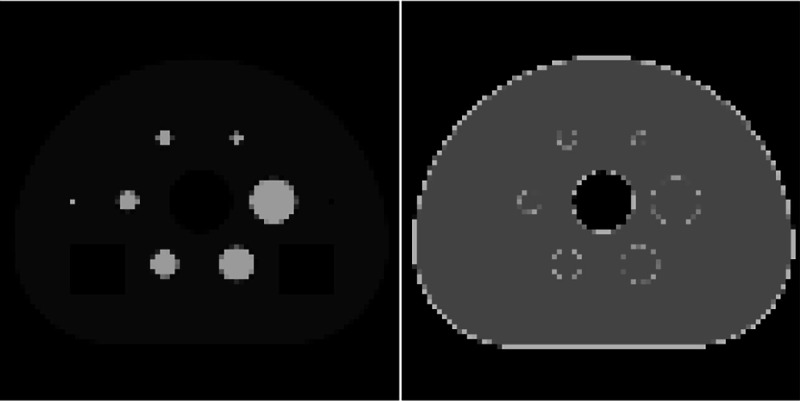
(Left) Slice number 40 from the University of Washington Digital Reference Object: activity image in arbitrary units, and (Right) attenuation map displayed in the gray scale window [**0**.**075**, **0**.**115**] cm^−**1**^.

The results demonstrating the ADMM-SAA algorithm are all derived from a 2D simulation using the digital reference object (DRO) shown in Fig. 1 [25]. This digital phantom is binned down to 128×128 image array with physical dimension 30×30 cm^2^. The LORs are arranged in a 2D parallel-beam geometry with 128 views covering a π radian arc, and 128 parallel rays being measured per view with a spacing of 0.234 cm (30/128 ≈ 0.234). The TOF FWHM is taken to be 9 cm, which corresponds to a timing resolution of approximately 600 picoseconds. The spacing between TOF window samples is 4.5 cm, and a total of ten TOF samples are taken per LOR. For the image reconstruction, both the attenuation and activity images are represented on a 128×128 grid. The purpose of the presented results is to demonstrate usage of the ADMM-SAA algorithm and the impact of the TV constraints on the reconstruction of the activity and attenuation.

For the following results, the biconvex alternation loop at line 10 of Algorithm 1 is run for $N_{y}=100$ iterations, and the Newton solver at line 15 is run for $N_{\text{newt}}=10$ iterations. With both of these loop settings, Eq. (22) is solved accurately in a numerical sense. Even with the inner loops being executed with such high iteration numbers, the efficiency of the whole biconvex alternation loop is still high, because all of the computations separate across the vector components. The computational effort for the biconvex alternation loop is $O\left(N_{y}\cdot N_{\text{TOF}}\cdot N_{\text{views}\;}\cdot \sqrt{N_{\text{pix}\;}}\right)$ (the Newton loop does not increase the order of this loop because it involves the attenuation sinogram only), and by comparison, computing TOF projection, *Tλ*, is $O\left(N_{\text{TOF}}\cdot N_{\text{views}\;}\cdot N_{\text{pix}\;}\right)$, where $N_{\text{pix}}$ is the total number of pixels and $N_{\text{views}}$ is the number of projection angles. For the small 128×128 images of this study the biconvex loop is of the same order at TOF projection because $N_{y}\approx \sqrt{N_{\text{pix}}}$, but as the data and image size increase, TOF projection becomes the more burdensome computation. It is also possible, in practice, to reduce $N_{y}$ and $N_{\text{newt}}$ and work with inexact solution of Eq. (22) but we do not investigate this option in this work.

### A. SAA from noiseless data

**Fig. 2.**
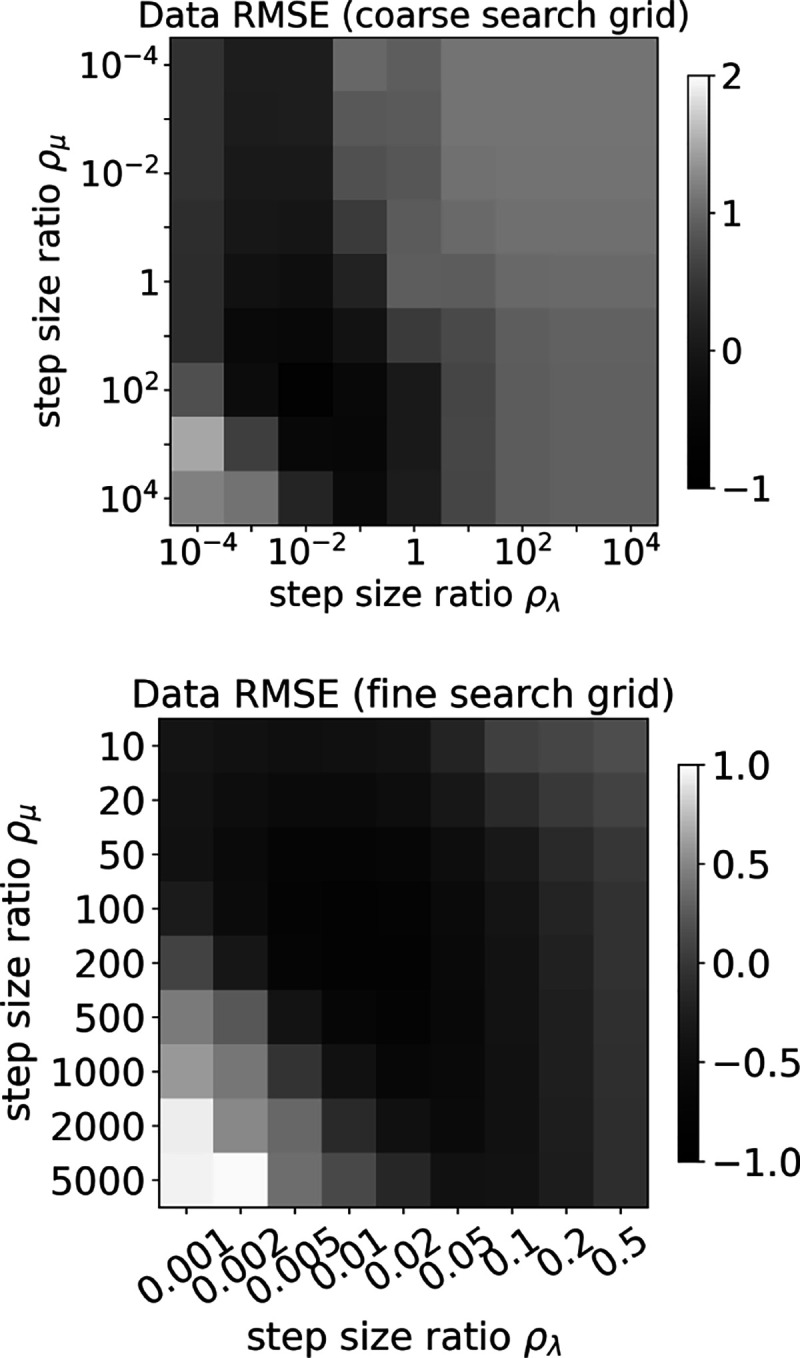
(Top) Coarse grid search, where 81 pairs of step size ratios are tested. The quantity **log**_**10**_( data RMSE ) is displayed in gray scale. (Bottom) Fine grid search, also testing 81 pairs of $\rho_{\lambda }$ and $\rho_{\mu }$, where **log**_**10**_( data RMSE ) is displayed. The minimum data RMSE value occurs at $\rho_{\lambda }=0.01$ and $\rho_{\mu }=100$.

Image reconstruction is performed on noiseless data using the mean counts as the measured data using Algorithm 1, which aims to solve the optimization problem in Eq. (20). Executing Algorithm 1 requires that the step size parameters be specified, which is done uniquely by setting values for the step size ratios $\rho_{\lambda }$ and $\rho_{\mu }$ from Eqs. (23) and (24), respectively. Tuning the values for $\rho_{\lambda }$ and $\rho_{\mu }$ is crucial for optimizing the algorithm efficiency, and this is one of the purposes of starting with noiseless data. A grid search is performed on the step size ratios, executing 100 iterations for each setting. Because noiseless consistent data are used, the discrepancy between the estimated and input data is zero at convergence. Accordingly, the data root mean square error (RMSE) as a measure of the progress toward the optimization solution, and we determine the step size ratios as the ones that minimize data RMSE after 100 iterations. Two grid searches are performed, over a coarse and fine grid, and the results of this search are shown in Fig. 2.

**Fig. 3.**
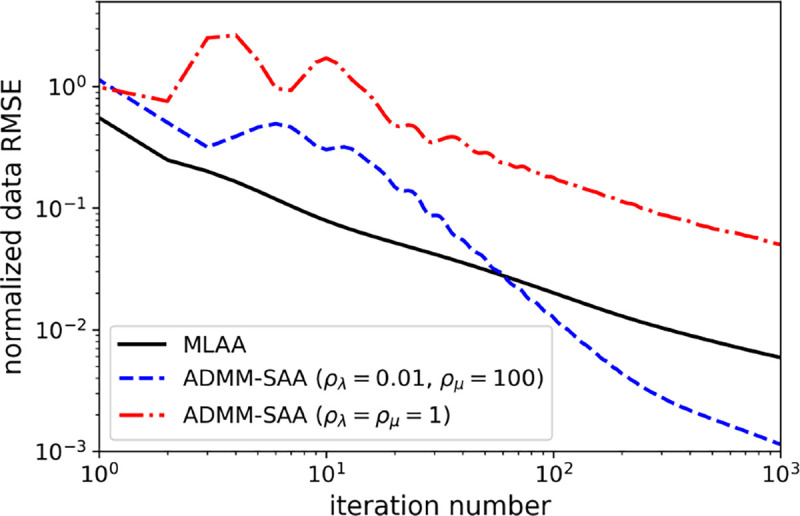
Comparison of data RMSE convergence for MLAA, ADMM-SAA with non-optimal settings $\rho_{\lambda }=\rho_{\mu }=1$, and ADMM-SAA with the tuned values $\rho_{\lambda }=0.01$ and $\rho_{\mu }=100$.

The utility of the step size parameter tuning and comparison with MLAA on data RMSE convergence is shown in Fig. 3. If the step size parameters are chosen so that σ and τ are equal, i.e. ρ=1, it is clear from the plotted data RMSE that convergence can be quite sub-optimal. Tuning $\rho_{\lambda }$ and $\rho_{\mu }$ results in two orders of magnitude smaller data RMSE after running ADMM-SAA for 1000 iterations. For reference, the convergence of MLAA on data RMSE is also included, and it can be seen that ADMM-SAA can be tuned so that it is more efficient than MLAA for the particular conditions of the 2D TOF-PET simulation. We note that both ADMM-SAA and MLAA are optimizing the same objective function. There is some variability in the presented technique for tuning $\rho_{\lambda }$ and $\rho_{\mu }$. Small variation in the results are expected depending on the scanned object, the number of ADMM-SAA iterations, the metric used to quantify convergence, and discretization in the grid search. This variability, however, does not have a large impact on the algorithm efficiency as can be seen in the broad minimum of the fine grid search in Fig. 3. In the following studies, using various forms of ADMM-TVSAA, the same step size ratio parameter settings are used.

For the next set of results with noiseless, consistent TOF-PET data, the recovery of the activity and attenuation images is investigated. Specifically, ADMM-TVSAA is employed to study the effectiveness for solving the associated inverse problem by imposing TV constraints on the activity alone, the attenuation alone, and both activity and attenuation. For this study, when TV constraints are imposed, the ground truth values are used as the corresponding constraint values. This tests the ability of ADMM-TVSAA to recover the ground truth images under ideal conditions. Turning off a TV constraint is achieved by setting the corresponding constraint value γ to a value much larger than the ground truth.

**Fig. 4.**
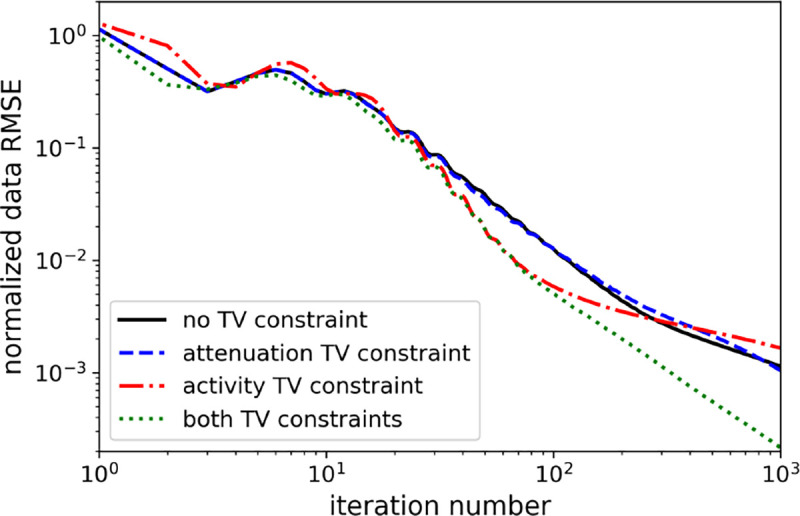
Comparison of TV-constraints on normalized data RMSE convergence.

**Fig. 5.**
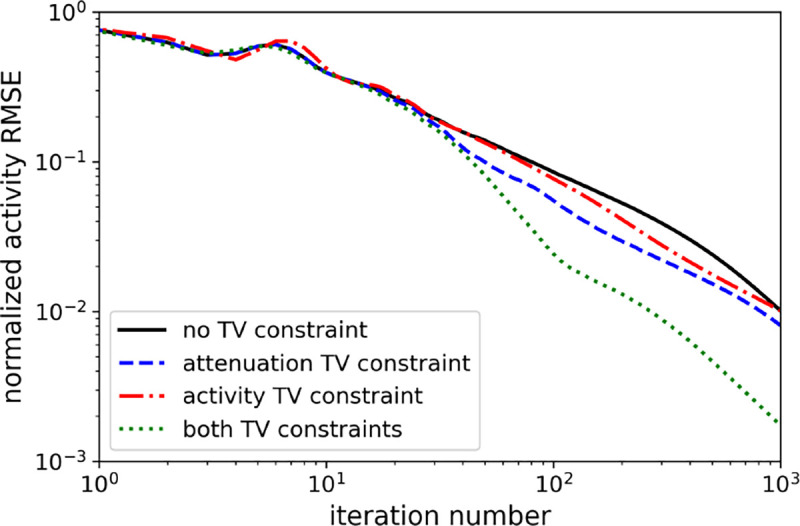
Comparison of TV-constraints on the normalized activity RMSE convergence.

**Fig. 6.**
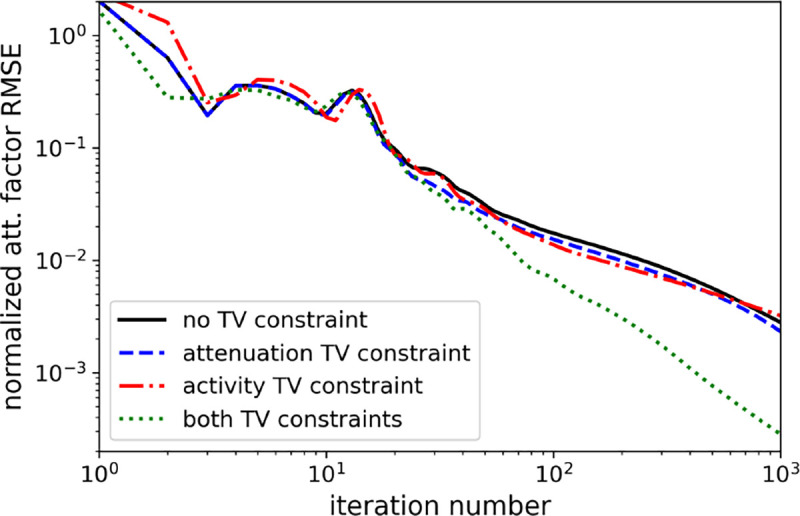
Comparison of TV-constraints on normalized attenuation factor RMSE convergence.

**Fig. 7.**
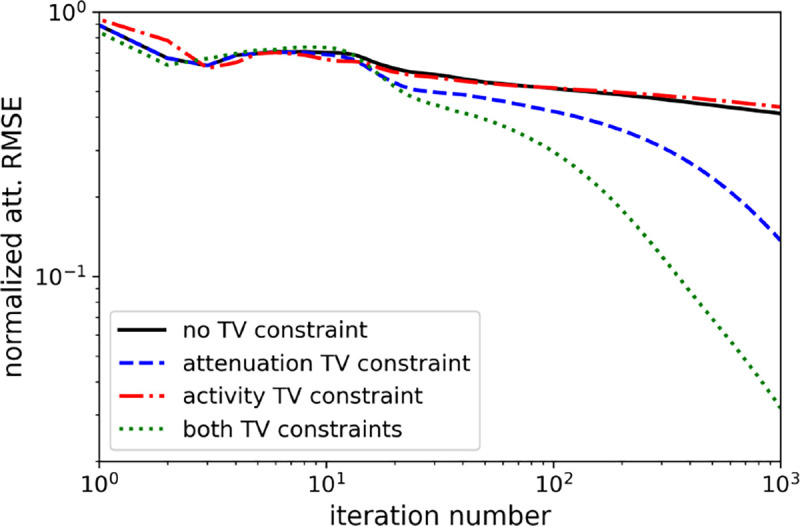
Comparison of TV-constraints on normalized attenuation RMSE convergence.

The results for the use of ADMM-TVSAA with different TV constraints active are shown in Figs. 4, 5, 6, and 7, which show RMSE measures for the TOF-PET data, activity, attenuation factor exp[−Pμ], and attenuation map, respectively. The reason why the attenuation factor RMSE is plotted is that it is the activity image that is the desired quantity that needs to be recovered. The attenuation map only needs to be recovered to the extent that the attenuation factor in the measurement model in Eq. (1) is accurate. The RMSE curves in Figs. 4–6 all show a downward trend over the 1000 iterations of ADMM-TVSAA. The result corresponding to the use of both activity and attenuation TV constraints converges more quickly than the other conditions; nevertheless all of the cases converge the activity image RMSE to better than 10^−2^. At this RMSE value the reconstructed activity is not visually distinguishable from the true activity.

**Fig. 8.**
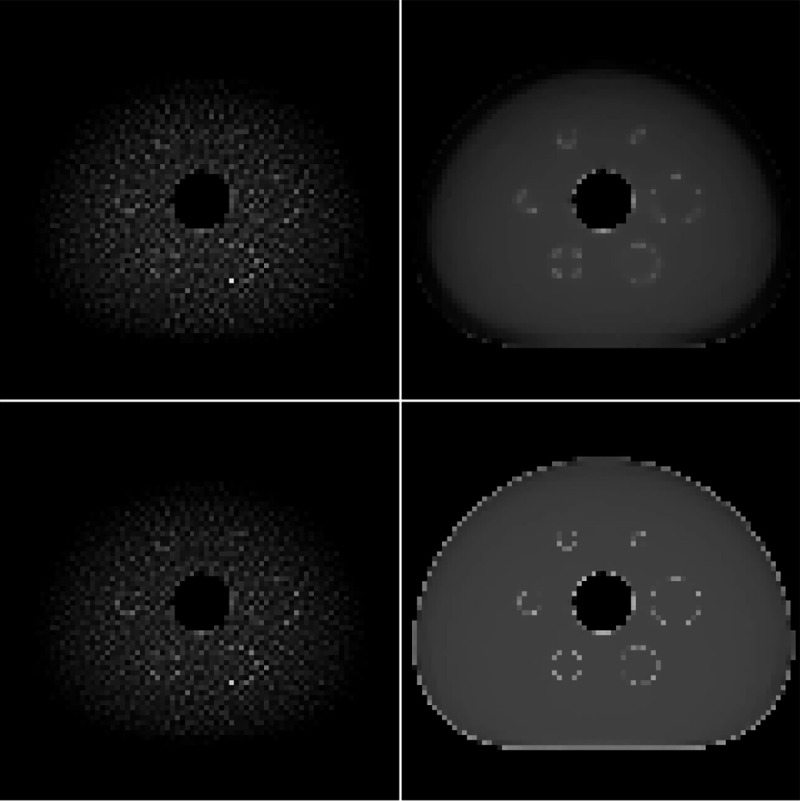
Comparison of TV-constraints on reconstructed attenuation maps: no TV constraints (Upper Left), attenuation TV constraint only (Upper Right), activity TV constraint only (Lower Left), and attenuation and activity TV constraints (Lower Right). Gray scale window is [**0**.**075**, **0**.**115**] cm^−**1**^.

The results for the attenuation RMSE convergence tell a different story. For this metric, use of both constraints yields the fastest convergence, and use of a TV constraint on the attenuation map results in faster convergence than the remaining two cases. The attenuation maps after 1000 iterations of ADMM-TVSAA are shown in Fig. 8, where it is seen that the use of both TV constraints yields an accurate estimate of the ground truth attenuation map. From these results, however, we cannot rule out that the other constraint combinations will yield an accurate attenuation map estimate; all of the RMSE curves do show a downward trend and it may be that after sufficient iteration numbers are reached that the attenuation map is indeed recovered.

### B. SAA from noisy data

**Fig. 9.**
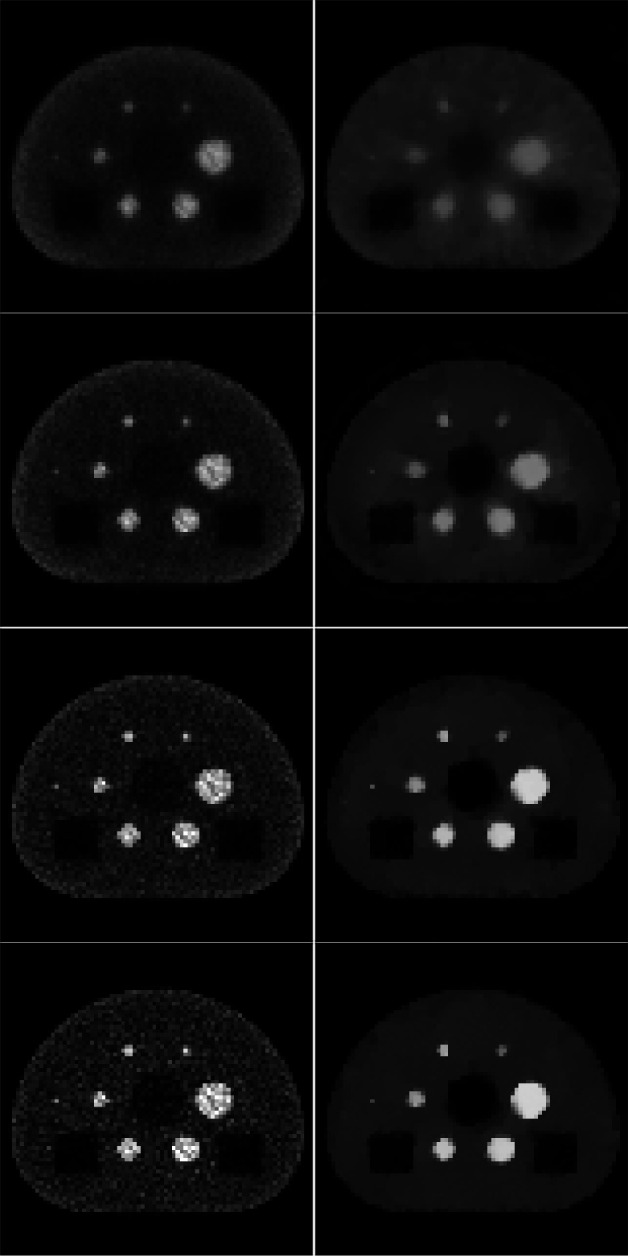
Reconstructed images for (left column) MLAA and (right column) TV-constrained ADMM-SAA ($\gamma_{\lambda }=1.0$, $\gamma_{\mu }=1.0$) from noisy data. The rows correspond to the images at 10, 20, 50, and 100 iterations going from top to bottom. The TOF-PET simulation assumes Poisson distributed noise with a total of 10^**6**^ measured coincidence counts.

The next set of studies focus on SAA with noisy data. Noise realizations are obtained by scaling the mean TOF-PET data so that the total number of measured coincidences is 10^6^; the realization is then obtained by selecting a number of detected coinidences for each LOR drawn from a Poisson distribution. To demonstrate the use of ADMM-TVSAA, the MLAA algorithm is used as a reference and the activity image estimates are displayed as a function of iteration number. The results for SAA from a single noise realization are shown in Fig. 9, where the TV constraint values are given in terms of the ground truth values and activity estimates are shown for iteration numbers between 10 and 100. When ground truth TV constraint values are not available, a validation technique can be exploited to discover the subject’s TV values as discussed in Ref. [26]. Visually, the images in Fig. 9 show low bias recovery of the activity distribution by iteration number 100 with a much smaller noise amplitude for ADMM-TVSAA as compared to the MLAA result.

**Fig. 10.**
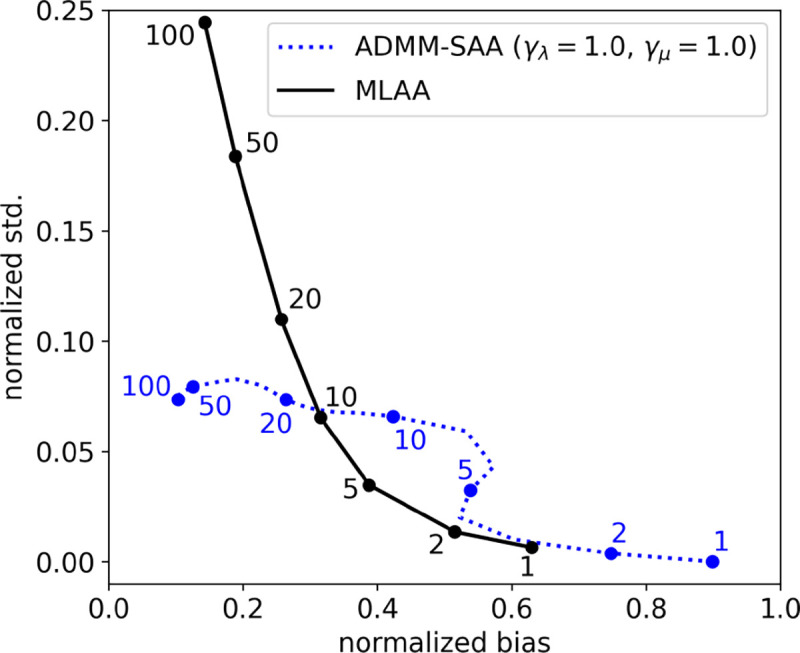
Normalized standard deviation (std.) versus normalized bias as a function of iteration number computed empirically from 100 noise realizations for MLAA and TV-constrained SAA. The labeled dots indicate the iteration numbers for the respective algorithm curves. For TV-constrained ADMM-SAA, curves are shown for activity and attenuation TV constraints set to $\gamma_{\lambda }=1.0$ and $\gamma_{\mu }=1.0$, respectively. The constraint values are given as a fraction of the ground truth TV values.

For a quantitative bias-variance analysis, MLAA and ADMM-TVSAA are used to perform SAA on an ensemble of 100 noise realizations of TOF-PET data. The mean and pixel standard deviation for both MLAA and ADMM-TVSAA are computed and plotted in Fig. 10 as a function of iteration number. The use of the TV-constraints, allows ADMM-TVSAA to achieve activity estimates with low bias and variance as compared with basic maximum-likelihood estimation as implemented with MLAA. Again, the MLAA result is used only as a reference since it is the algorithm standard for SAA in TOF-PET.

**Fig. 11.**
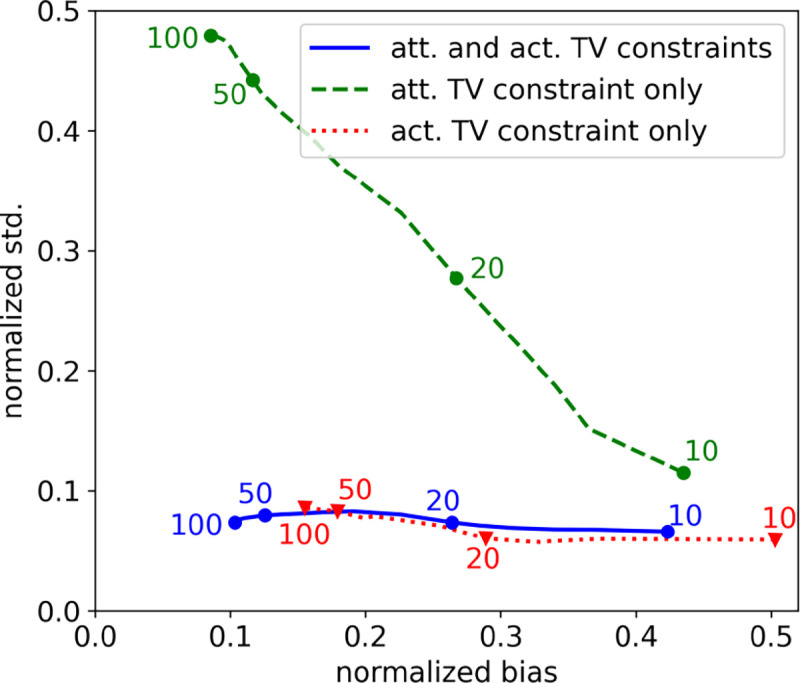
Normalized standard deviation (std.) versus normalized bias as a function of iteration number computed empirically from 100 noise realizations for SAA with different TV constraint combinations. The dots indicate the iteration numbers 10, 20, 50, and 100 for the respective algorithm curves. In each case, the constraint value is set to the ground truth TV value.

For the final study, bias-variance analysis is performed to gain an understanding of the impact of the activity and attenuation TV constraints. The image bias and pixel standard deviation are plotted in Fig. 11 as a function of iteration number for various combinations of imposing TV constraints. The curve for the use of a TV constraint on the activity alone shows the effectiveness of this constraint in controlling the image variance. The use of the TV constraint on the attenuation map alone results in increasing image variance as a function of iteration number, but there is a reduction in the image bias as compared with use of an activity TV constraint alone. Imposing both activity and attenuation constraints yields benefit in reducing image variance and bias as compared with the other two cases.

## IV. Discussion and conclusion

In this work, an ADMM framework is developed that can be applied to nonsmooth and nonconvex optimization problems that arise in imaging. The particular form of nonconvexity addressed is when the optimization problem has a biconvex structure. The imaging problem posed by simultaneous estimation of the activity and attenuation (SAA) in time-of-flight positron emission tomography (TOF-PET) has such a structure. Using this ADMM framework, a limited study on the impact of total variation (TV) constraints on the activity and attenuation for the SAA problem is presented. The use of both of these constraint is seen to help stabilize the SAA inverse problem as demonstrated by the noiseless results. When using noisy data, the TV constraints help to reduce image bias and variance as compared with use of maximum likelihood estimation alone. The shown results are intended to show the potential in solving the SAA inverse problem with the use of TV constraints on the activity and attenuation and to demonstrate the ADMM-TVSAA algorithm. While we have shown results only for ADMM-TVSAA, the ADMM framework is easily extended to include other nonsmooth, convex terms. Further study varying the test phantom and TOF-PET setup are needed to obtain a more comprehensive picture of the SAA inverse problem.

## Supplementary Material

Supplement 1

## Data Availability

The implementation of the algorithms, which are presented in this article, and the code, which generates the figures, are available at: https://github.com/zhimeir/saa_admm_paper.
