# Simultaneous activity and attenuation estimation in TOF-PET with TV-constrained nonconvex optimization

**Published:** 2024-02-09

**Authors:** Zhimei Ren, Emil Y. Sidky, Rina Foygel Barber, Chien-Min Kao, Xiaochuan Pan

**Affiliations:** Dept. of Statistics and Data Science, University of Pennsylvania; Dept. of Radiology, University of Chicago; Dept. of Statistics, University of Chicago; Dept. of Radiology, University of Chicago; Dept. of Radiology, University of Chicago

**Keywords:** Image reconstruction in TOF-PET, simultaneous activity/attenuation estimation, large-scale nonconvex optimization, alternating direction method of multipliers

## Abstract

An alternating direction method of multipliers (ADMM) framework is developed for nonsmooth biconvex optimization for inverse problems in imaging. In particular, the simultaneous estimation of activity and attenuation (SAA) problem in time-of-flight positron emission tomography (TOF-PET) has such a structure when maximum likelihood estimation (MLE) is employed. The ADMM framework is applied to MLE for SAA in TOF-PET, resulting in the ADMM-SAA algorithm. This algorithm is extended by imposing total variation (TV) constraints on both the activity and attenuation map, resulting in the ADMM-TVSAA algorithm. The performance of this algorithm is illustrated using the penalized maximum likelihood activity and attenuation estimation (P-MLAA) algorithm as a reference. Additional results on step-size tuning and on the use of unconstrained ADMM-SAA are presented in the previous arXiv submission: arXiv:2303.17042v1.

## I. Introduction

Nuclear medicine imaging modalities such as single-photon emission computed tomography (SPECT) and positron emission tomography (PET) require the input of a gamma ray attenuation map for quantitatively accurate imaging. The combination of nuclear medicine imaging with other image modalities such as X-ray computed tomography (CT) [1], [2] or magnetic resonance imaging (MRI) [3] provides a means for estimating the necessary attenuation map. There are, however, challenges in the separate attenuation map estimation. Use of CT-based attenuation maps requires extrapolation of the photon attenuation map from the diagnostic X-ray energy range to 511 keV and registration of the PET and CT imaging, which can be particularly difficult in the presence of motion [4]. The use of MRI to estimate a synthetic CT image is further complicated by the fact that bone and air have similar gray values in MRI while bone has a significantly higher attenuation coefficient for gamma rays.

To avoid a separate measurement for obtaining the gamma ray attenuation map, a long-standing inverse problem of interest has been to simultaneously estimate the attenuation and activity distributions from emission data alone [5], [6]. To address simultaneous activity and attenuation (SAA) estimation, Nuyts *et al*. [6] use maximum likelihood to invert the algebraic SAA model, and they find that accurate activity distributions can be recovered by appropriately regularizing the attenuation map. The regularization involves the use of Gibbs and intensity priors on the attenuation distribution that encourage local smoothness and clustering of values around known attenuation values for tissues in the scanned subject. Another interesting result for the SAA problem is obtained in considering time-of-flight positron emission tomography (TOF-PET) [7]. Defrise *et al*. [8] exploit an analytic range condition [9], [10] for the continuous TOF-PET model and obtain a uniqueness result that the attenuation factor and activity can be determined up to a multiplicative constant. Returning to the SAA algebraic model for TOF-PET, a comprehensive study of this inverse problem using maximum likelihood estimation is presented in Rezaei *et al*. [11], where it is found that the activity and attenuation maps can be recovered if the timing resolution of the TOF measurements is sufficiently high and if support constraints are exploited. We note an intriguing extension of the SAA problem where the background radiation from Lutetium-176, present in PET scintillators composed of either lutetium oxyorthosilicate (LSO) or lutetium-yttrium orthosilicate (LYSO), is exploited to provide additional information on the subject’s attenuation map without the need for a separate scan [12]. Also, in the context of PET/MRI, anatomical information from standard MRI protocols can be used as a prior to inform the SAA estimation without the need for dedicated pulse sequences need for MR-based attenuation correction [13].

In this work, we seek to build off of Ref. [11] and develop an image reconstruction framework for the SAA problem in TOF-PET that can incorporate nonsmooth, convex constraints in the maximum likelihood estimation. Such constraints can help to achieve stable inversion of the SAA estimation problem. Of particular interest, here, is the use of total variation (TV) constraints on both activity and attenuation distributions. We have previously exploited such constraints in the context of nuclear medicine imaging; in Refs. [14] and [15] TV constraints are exploited to enable sparse-data sampling configurations in SPECT and PET, respectively. In Ref. [16], a similar methodology is used for image reconstruction in low-count list-mode TOF-PET.

The image reconstruction algorithms developed in Refs. [14]–[16] are all instances of a general primal-dual (PD) solver for nonsmooth convex optimization developed by Chambolle and Pock [17], [18]. The optimization problem posed by applying TV-constraints to the SAA estimation problem, however, is nonsmooth and nonconvex. In our recent work, we develop a framework for such problems in imaging, where the optimization can be split into convex terms plus differentiable terms that are possibly nonconvex [19]. This framework is based on the alternating direction method of multipliers (ADMM) [20] in a way that is closely related to the PD algorithm. This framework has been successfully applied to the nonsmooth and nonconvex optimization problem that arises in spectral computed tomography (CT) when the spectral response of the measurement is included in the data model [21]. Here, we modify this framework to address biconvex optimization and apply it to the SAA estimation problem with convex constraints. The SAA data model and imaging problem are specified in Sec. II, where we then develop an ADMM algorithm to solve the associated optimization problem. As the focus of this work is mainly on the SAA inverse problem, we conduct a number of studies on noiseless TOF-PET data in Sec. III that explore the range of TOF-PET parameters that allow exact recovery of activity and attenuation factors. Also presented in this section are results with noisy data that demonstrate the stability of the proposed algorithm. In Sec. V the results are discussed and the conclusions of the work are given.

## II. Image reconstruction model and algorithms

In presenting the SAA algorithm TOF-PET, we consider a two dimensional (2D) simulation where the lines-of-response (LORs) are organized in parallel-ray fashion and are specified in the same way that the 2D Radon transform is parameterized. For the TOF-PET model, the Radon transform is modified by including weighted line-integration that accounts for TOF information that helps to localize the positron-electron annihilation along a given LOR. After specifying the TOF-PET data model, the MLAA algorithm from Rezaei *et al*. [11] is briefly summarized. We then present the nonconvex ADMM algorithm that performs SAA estimation with nonsmooth convex constraints.

### A. TOF-PET modelling

The measurement model for the mean data in TOF-PET is

(1) $$c_{i\ell }=\text{exp}\left[-P_{\ell }^{⊤}\mu \right]\cdot T_{i\ell }^{⊤}\lambda ,$$

where λ and μ are the unknown activity and attenuation maps, respectively; $T_{i\ell }$ is the TOF sensitivity image for TOF window i, LOR ℓ; $P_{\ell }$ is the X-ray projection matrix sensitivity image for LOR ℓ. For defining the TOF projection matrix T, the TOF window sensitivity along the LOR is specified as

$$w_{i}\left(t\right)=\text{exp}\left[-{\left(t-t_{i}\right)}^{2}/\left(2\sigma_{\text{TOF}}\right)\right],$$

where the sampling along the LOR is half of the full-width-half-maximum (FWHM) of this Gaussian distribution

$$\Delta t=t_{i+1}-t_{i}=\text{FWHM}/2=\sqrt{2\text{ log }2}\cdot \sigma_{\text{TOF}}.$$

For this work, scatter coincidences and random events are not considered.

### B. Imaging model based on nonconvex optimization

We consider performing SAA using likelihood maximization, where the measured coincidence count data are assumed to follow a multivariate, mutually independent Poisson distribution

$$C_{i\ell }\sim \text{Poisson}\left(c_{i\ell }\right).$$

Equivalently, this estimation is performed by minimization of the negative log-likelihood,

(2) $$l\left(\lambda ,\mu \right)=\underset{i\ell }{\sum }\left\{c_{i\ell }-C_{i\ell }\cdot \text{log }c_{i\ell }\right\}=\;\underset{i\ell }{\sum }\left\{\text{exp}\left(-P_{\ell }^{⊤}\mu \right)\cdot T_{i\ell }^{⊤}\lambda -C_{i\ell }\cdot \left(-P_{\ell }^{⊤}\mu +\text{log}\left(T_{i\ell }^{⊤}\lambda \right)\right)\right\}.$$

The optimization problem of interest is

(3) $$\lambda ,\mu =\underset{\lambda ,\mu }{\text{arg min}}\left\{l\left(\lambda ,\mu \right)|1^{⊤}\lambda =N_{\text{total}},\;\lambda ,\mu \geq 0\right\},$$

where l is the negative log-likelihood in Eq. (2); 1 is a vector of size λ with unit entries so that $1^{⊤}\lambda$ is equivalent to summation over λ; and $N_{\text{total}}$ is the total number of annihilations. The constraint on the total number of annihilations is used to overcome the constant ambiguity in the SAA estimation problem [8]. This constraint is enforced in this work instead of the object support constraint investigated in Rezaei *et al*. [11].

### C. Summary of MLAA

To solve this imaging model, Rezaei *et al*. [11] developed the MLAA algorithm. For completeness, we write the MLAA update steps including a minor modification in Eq. (6) that accommodates the constraint on the total number of annihilations:

(4) $$a_{\ell }=\text{exp}\left[-\underset{k}{\sum }P_{\ell k}\mu_{k}\right]\;\forall \ell ,$$

(5) $$\lambda_{k}\leftarrow \frac{\lambda_{k}}{\sum_{i\ell }a_{\ell }T_{i\ell k}}\underset{i\ell }{\sum }\left\{T_{i\ell k}\left(\frac{C_{i\ell }}{\sum_{k^{\prime }}T_{i\ell k^{\prime }}\lambda_{k^{\prime }}}\right)\right\}\;\forall k,$$

(6) $$\lambda \leftarrow \lambda \left(\frac{N_{\text{total}}}{\sum_{k}\lambda_{k}}\right),$$

(7) $$\mu_{k}\leftarrow \mu_{k}+\frac{\sum_{i\ell k^{\prime }}P_{\ell k}\left(a_{\ell }T_{i\ell k^{\prime }}\lambda_{k^{\prime }}-C_{i\ell }\right)}{\sum_{i\ell k^{\prime }}P_{\ell k^{\prime }}P_{\ell k}a_{\ell }\sum_{k^{\prime \prime }}T_{i\ell k^{\prime \prime }}\lambda_{k^{\prime \prime }}}\;\forall k,$$

(8) $$\mu_{k}.\leftarrow \text{pos}\left(\mu_{k}\right)\;\forall k.$$

The MLAA algorithm essentially alternates between updating λ with a Poisson likelihood EM step and μ with a Poisson transmission likelihood optimization step. In this MLAA implementation the extra update step in Eq. (6) enforces the constraint on the total number of annihilations, and Eq. (8) performs non-negativity projection, where negative values of μ are set to zero. For MLAA the activity λ should have a strictly positive initialization, and this quantity will remain non-negative during the iteration.

Early stopping of the iteration is the primary means of performing regularization with MLAA, but explicit regularization can also be included with the use of Gibbs smoothing [22]–[24]. In this work, we develop a framework for SAA which can include nonsmooth regularization.

### D. ADMM for nonsmooth and biconvex optimization

The general convex optimization problem that ADMM solves takes the form

$$\underset{x,y}{\text{min}}\left\{f\left(x\right)+g\left(y\right)|Ax+By=c\right\},$$

where f and g are convex and possibly non-smooth functions; A and B are linear operators; x,y and c are vectors. The steps of the ADMM algorithm are

(9) $$x\leftarrow \underset{x^{\prime }}{\text{arg min}}\left\{f\left(x^{\prime }\right)+u^{⊤}Ax^{\prime }+\frac{1}{2}{\left\|Ax^{\prime }+By-c\right\|}_{\Sigma }^{2}+\frac{1}{2}{\left\|x^{\prime }-x\right\|}_{H_{f}}^{2}\right\}$$

(10) $$y\leftarrow \underset{y^{\prime }}{\text{arg min}}\left\{g\left(y^{\prime }\right)+u^{⊤}By^{\prime }+\frac{1}{2}{\left\|Ax+By^{\prime }-c\right\|}_{\Sigma }^{2}+\frac{1}{2}{\left\|y^{\prime }-y\right\|}_{H_{g}}^{2}\right\}$$

(11) u←u+Σ(Ax+By−c),

where Σ, $H_{f}$, and $H_{g}$ are symmetric positive definite, and $∥v{∥}_{M}^{2}\equiv v^{⊤}Mv$ for any symmetric positive definite matrix M. Because optimizing the TOF-PET likelihood for SAA is a non-convex optimization problem, the ADMM algorithm does not directly apply. One strategy to adapt ADMM to SAA is to base the ADMM steps on a series of successive convex approximations as developed by Chun *et al*. [25] using the separable quadratic surrogates (SQS) method. In the present work, we develop an alternative form of ADMM that directly applies to SAA, exploiting the biconvex structure of the TOF-PET likelihood function; i.e. fixing either λ or μ, the likelihood is a convex function in the other variable.

The ADMM algorithm can be modified to accommodate a biconvex function, and we consider the case that only g is a biconvex function

$$g\left(y\right)=g\left(y_{1},y_{2}\right),$$

where y is the concatenation of $y_{1}$ and $y_{2}$; and $g\left(y_{1},\cdot \right)$ and $g\left(\cdot ,y_{2}\right)$ are convex functions for fixed $y_{1}$ and $y_{2}$, respectively. To accommodate the biconvexity of g, the second update equation, Eq. (10), is replaced by an inner iteration with the following update equations

(12) $$y_{1}\leftarrow \underset{y_{1}^{\prime }}{\text{arg min}}\{g\left(y_{1}^{\prime },y_{2}\right)+u^{⊤}B\left(y_{1}^{\prime },y_{2}\right)+\frac{1}{2}{\left\|Ax+B\left(y_{1}^{\prime },y_{2}\right)-c\right\|}_{\Sigma }^{2}+\frac{1}{2}{\left\|\left(y_{1}^{\prime },y_{2}\right)-\left(y_{1},y_{2}\right)\right\|}_{H_{g}}^{2}\}$$

(13) $$y_{2}\leftarrow \underset{y_{2}^{\prime }}{\text{arg min}}\left\{g\left(y_{1},y_{2}^{\prime }\right)+u^{⊤}B\left(y_{1},y_{2}^{\prime }\right)+\frac{1}{2}{\left\|Ax+B\left(y_{1},y_{2}^{\prime }\right)-c\right\|}_{\Sigma }^{2}+\frac{1}{2}{\left\|\left(y_{1},y_{2}^{\prime }\right)-\left(y_{1},y_{2}\right)\right\|}_{H_{g}}^{2}\right\}.$$

The inner loop consists of alternating between Eqs. (12) and (13) for a predetermined number of iterations $N_{y}$, where $N_{y}\geq 1.$ After the inner loop is completed, the ADMM iteration continues with Eq. (11) after the following assignment

$$y=\left(y_{1},y_{2}\right).$$

This inner loop, specified in Eqs. (12) and (13), is computationally efficient if multiplication by the matrix B is efficient; this is the case in our application because we consider B=I where I is the identity matrix. Note that multiplication by A is not performed within this inner iteration because the matrix A only appears in the term Ax which is computed before entering the inner loop.

### E. ADMM for large-scale tomographic image reconstruction

For the large-scale optimization problems that arise in tomographic image reconstruction, the update step in Eq. (9) can be problematic because of the term Ax, which appears in the minimization over x. The matrix A usually contains the system matrix for the imaging model, and computation of Ax can be expensive particularly for 3D imaging; thus numerical solution of Eq. (9) may not be feasible. This “expensive inner loop” problem can be circumvented by linearization, i.e. by including the additional term $\frac{1}{2}{\left\|x^{\prime }-x\right\|}_{H_{f}}^{2}$ in Eq. (9) [19], [26], resulting in an algorithm closely related to the primal-dual (PD) algorithm of Chambolle and Pock [17], [18]. Considering only scalar step size parameters, i.e.

Σ=σI,

the metric $H_{f}$ in Eq. (9) is set to

(14) $$H_{f}=I/\tau -\sigma A^{⊤}A.$$

This choice cancels the $Ax^{\prime }$ term in Eq. (9), and the requirement that $H_{f}$ be positive definite yields a constraint on the step sizes σ and τ. In the context of the image reconstruction problem, we also have

$$H_{g}=0;\;B=-I;\;c=0.$$

The ADMM generic optimization problem becomes

(15) $$\underset{x,y}{\text{min}}\left\{f\left(x\right)+g\left(y\right)|Ax-y=0\right\},$$

and the algorithm for convex optimization is then specified by the following update equations

(16) $$x\leftarrow \underset{x^{\prime }}{\text{arg min}}\left\{f\left(x^{\prime }\right)+{x^{\prime }}^{⊤}A^{⊤}\left(u+\sigma \left(Ax-y\right)\right)+\frac{1}{2\tau }{\left\|x^{\prime }-x\right\|}^{2}\right\}$$

(17) $$y\leftarrow \underset{y^{\prime }}{\text{arg min}}\left\{g\left(y^{\prime }\right)-u^{⊤}y^{\prime }+\frac{\sigma }{2}{\left\|Ax-y^{\prime }\right\|}^{2}\right\}$$

(18) u←u+σ(Ax−y).

Aside from minor details, this set of update equations is equivalent to the PD algorithm, but as a starting point to modify the update steps for non-convex optimization, this form is more convenient because both f and g functions appear directly in the updates. In contrast, the PD algorithm dualizes g and the convex conjugate $g^{⋆}$ is needed. If it is desired to apply PD to non-convex g, figuring out what to put in place of $g^{⋆}$, while possible [27], adds another layer of complication to the algorithm development.

The modification of the linearized ADMM updates for addressing the case where g is biconvex replaces Eq. (17) with inner loop update equations

(19) $$y_{1}=\underset{y_{1}^{\prime }}{\text{arg min}}\left\{g\left(y_{1}^{\prime },y_{2}\right)-u^{⊤}\left(y_{1}^{\prime },y_{2}\right)+\frac{\sigma }{2}{\left\|Ax-\left(y_{1}^{\prime },y_{2}\right)\right\|}^{2}\right\}$$

(20) $$y_{2}=\underset{y_{2}^{\prime }}{\text{arg min}}\{g\left(y_{1},y_{2}^{\prime }\right)-u^{⊤}\left(y_{1},y_{2}^{\prime }\right)\;+\frac{\sigma }{2}{\left\|Ax-\left(y_{1},y_{2}^{\prime }\right)\right\|}^{2}\}.$$

Convergence of this modified ADMM algorithm for biconvex functions is not theoretically guaranteed and thus convergence is demonstrated empirically.

### F. ADMM for SAA in TOF-PET

The instantiation of ADMM for SAA estimation by minimization of the negative log-likelihood is covered here in detail. The optimization problem of interest, restated from Eq. (3), is

(21) $$\lambda ,\mu =\underset{\lambda ,\mu }{\text{arg min}}\left\{l\left(\lambda ,\mu \right)|1^{⊤}\lambda =N_{\text{total}},\;\lambda \geq 0,\;\mu \geq 0\right\}.$$

In this sub-section, we map this optimization problem on to the ADMM algorithm, derive the x-update and biconvex y-updates, and provide the pseudo-code for SAA estimation.

To map the optimization problem in Eq. (21) onto the generic ADMM optimization in Eq. (15), the primal, splitting, and dual variables x,y, and u, are respectively assigned as

$$x=\left(\begin{array}{l} \lambda \\ \mu \end{array}\right),\;y=\left(\begin{array}{l} y_{\lambda }\\ y_{\mu } \end{array}\right),\;u=\left(\begin{array}{l} u_{\lambda }\\ u_{\mu } \end{array}\right)\text{. }$$

The linear system A is assigned as

$$A=\left(\begin{array}{cc} T & 0\\ 0 & P \end{array}\right).$$

The convex function f is used to represent the non-negativity constraints and the constraint on the total number of annihilations by setting

(22) $$f\left(\lambda ,\mu \right)=\delta \left(1^{⊤}\lambda =N_{\text{total}}\right)+\delta \left(\lambda \geq 0\right)+\delta \left(\mu \geq 0\right),$$

where δ is the convex indicator function, which is zero if the conditional argument is true and infinity otherwise. The biconvex function g accounts for the negative log-likelihood objective function in Eq. (21)

(23) $$g\left(y_{\lambda },y_{\mu }\right)=L\left(y_{\lambda },y_{\mu }\right),\;L\left(y_{\lambda },y_{\mu }\right)=\underset{i\ell }{\sum }\left\{\text{exp}\left(-y_{\mu ,\ell }\right)\cdot y_{\lambda ,i\ell }-C_{i\ell }\cdot \left(-y_{\mu ,\ell }+\text{log}\left(y_{\lambda ,i\ell }\right)\right)\right\},$$

where

l(λ,μ)=L(Tλ,Pμ).

#### Parametrization of the step sizes:

Step size selection is a critical issue for first-order, large-scale optimization algorithms. There can be much flexibility in the step size selection, and it is important to select a minimal set of free parameters that are effective for algorithm efficiency but not too cumbersome in the tuning procedure. Because the system matrix A for SAA is block-diagonal, a slight generalization of the ADMM linearization is considered. The metric $H_{f}$ is written as

$$H_{f}=\left(\begin{array}{cc} H_{\lambda } & 0\\ 0 & H_{\mu } \end{array}\right),\;H_{\lambda }=\frac{I}{\tau_{\lambda }}-\sigma_{\lambda }T^{⊤}T,\;H_{\mu }=\frac{I}{\tau_{\mu }}-\sigma_{\mu }P^{⊤}P,$$

and the step size parameters are chosen according to

$$\sigma_{\lambda }\tau_{\lambda }=1/{\left\|T\right\|}_{2}^{2},\;\sigma_{\mu }\tau_{\mu }=1/{\left\|P\right\|}_{2}^{2},$$

where ${\left\|M\right\|}_{2}$ is the largest singular value of the matrix M. With four step size parameters and two equality constraints, there are two free step size parameters. Specifically, the step size ratios, $\rho_{\lambda }$ and $\rho_{\mu }$, are chosen to be the free parameters that need to be tuned:

(24) $$\sigma_{\lambda }=\rho_{\lambda }/{\left\|T\right\|}_{2},\;\tau_{\lambda }=1/\left(\rho_{\lambda }{\left\|T\right\|}_{2}\right),$$

(25) $$\sigma_{\mu }=\rho_{\mu }/{\left\|P\right\|}_{2},\;\tau_{\mu }=1/\left(\rho_{\mu }{\left\|P\right\|}_{2}\right).$$

Tuning of $\rho_{\lambda }$ and $\rho_{\mu }$ is a necessary step any time the T or P matrices are changed due to, for example, a change in scan configuration or sampling pattern.

#### The x-update:

For the SAA problem in TOF-PET the x-update in Eq. (16) splits into two optimization problems

(26) $$\lambda \leftarrow \underset{\lambda^{\prime }}{\text{arg min}}\left\{\lambda^{\prime ⊤}T^{⊤}\left(u_{\lambda }+\sigma_{\lambda }\left(T\lambda -y_{\lambda }\right)\right)+\frac{1}{2\tau_{\lambda }}\lambda^{\prime }-\lambda^{2}|1^{⊤}\lambda^{\prime }=N_{\text{total}},\lambda^{\prime }\geq 0\right\},$$

(27) $$\mu \leftarrow \underset{\mu^{\prime }}{\text{arg min}}\left\{\mu^{\prime ⊤}P^{⊤}\left(u_{\mu }+\sigma_{\mu }\left(P\mu -y_{\mu }\right)\right)+\frac{1}{2\tau_{\mu }}{\left\|\mu^{\prime }-\mu \right\|}^{2}|\;\mu^{\prime }\geq 0\right\},$$

where the indicator terms of the convex function f from Eq. (22) are incorporated as constraints in the λ- and μ-update equations. The optimization problem for the μ-update in Eq. (27) is solved by setting the gradient of the objective function to zero and solving for $\mu^{\prime }$, followed by a nonnegativity projection to enforce the constraint on μ,

(28) $$\mu \leftarrow \mu -\tau_{\mu }P^{⊤}{\bar{u}}_{\mu },\;{\bar{u}}_{\mu }=u_{\mu }+\sigma_{\mu }\left(P\mu -y_{\mu }\right),\;\mu \leftarrow \text{pos}(\mu ),$$

where the function pos(⋅) thresholds negative components of the argument to zero.

For the λ optimization problem in Eq. (26), completing the square in the quadratic objective function, rescaling the objective function, and ignoring the $\lambda^{\prime }$-independent term yields

(29) $$\lambda \leftarrow \underset{\lambda^{\prime }}{\text{arg min}}\left\{\frac{1}{2}{\left\|\lambda^{\prime }-\left(\lambda -\tau_{\lambda }T^{⊤}{\bar{u}}_{\lambda }\right)\right\|}^{2}|1^{⊤}\lambda^{\prime }=N_{\text{total}},\lambda^{\prime }\geq 0\right\},\;{\bar{u}}_{\lambda }=u_{\lambda }+\sigma_{\lambda }\left(T\lambda -y_{\lambda }\right).$$

This optimization problem is now in the form of a projection onto the positive simplex, for which an efficient algorithm is developed by Duchi *et al*. [28]. Computationally, the updates in λ and μ are the most expensive steps in the ADMM algorithm because they involve forward- and back-projection of μ and ${\bar{u}}_{\mu }$, respectively, in addition to TOF forward- and back-projection of λ and ${\bar{u}}_{\lambda }$, respectively.

#### The biconvex y-updates:

The g function in Eq. (23) is biconvex in that it is convex in $y_{\lambda }$ if $y_{\mu }$ is fixed and *vice versa*. Splitting up the g function over the two update equations in Eqs. (19) and (20) yields

(30) $$y_{\lambda }=\underset{y_{\lambda }^{\prime }}{\text{arg min}}\left\{\underset{i\ell }{\sum }\left(\text{exp}\left(-y_{\mu ,\ell }\right)\cdot y_{\lambda ,i\ell }^{\prime }-C_{i\ell }\cdot \text{log}\left(y_{\lambda ,i\ell }^{\prime }\right)\right)-u_{\lambda }^{⊤}y_{\lambda }^{\prime }+\frac{\sigma_{\lambda }}{2}{\left\|y_{\lambda }^{\prime }-T\lambda \right\|}^{2}\;|\;y_{\lambda }^{\prime }\geq 0\right\},$$

and

(31) $$y_{\mu }=\underset{y_{\mu }^{\prime }}{\text{arg min}}\left\{\underset{i\ell }{\sum }\left(\text{exp}\left(-y_{\mu ,\ell }^{\prime }\right)\cdot y_{\lambda ,i\ell }+C_{i\ell }\cdot y_{\mu ,\ell }^{\prime }\right)-u_{\mu }^{⊤}y_{\mu }^{\prime }+\frac{\sigma_{\mu }}{2}{\left\|y_{\mu }^{\prime }-P\mu \right\|}^{2}|\;y_{\mu }^{\prime }\geq 0\right\},$$

noting that the $\text{exp}\left(-y_{\mu }\right)\cdot y_{\lambda }$ term is the only one that mixes the $y_{\lambda }$ and $y_{\mu }$ variables and is therefore common to both minimization problems. In order for the biconvex alternation to converge it is necessary to introduce the non-negativity constraints on $y_{\lambda }^{\prime }$ and $y_{\mu }^{\prime }$. Physically, these constraints are redundant with the non-negativity constraints imposed on λ and μ; if these physical constraints are not used, it is still necessary to impose non-negativity constraints on Tλ and Pμ.

The minimization problems for the y-update are both separable over the components of $y_{\lambda }^{\prime }$ and $y_{\mu }^{\prime }$. The minimization over $y_{\lambda }^{\prime }$ in Eq. (30) is solved analytically by setting the gradient of the objective function to zero, yielding a quadratic equation when $C_{i\ell }>0$,

$$\sigma_{\lambda }y_{\lambda ,i\ell }^{2}-b_{i\ell }y_{\lambda ,i\ell }-C_{i\ell }=0,$$

where $b_{i\ell }=u_{\lambda ,i\ell }+\sigma_{\lambda }T_{i\ell }^{⊤}\lambda -\text{exp}\left(-y_{\mu ,\ell }\right)$, and a linear equation when $C_{i\ell }=0$,

$$\sigma_{\lambda }y_{\lambda ,i\ell }-b_{i\ell }=0.$$

For the $C_{i\ell }>0$ case, the non-negativity constraint on $y_{\lambda }^{\prime }$ is respected by selecting the non-negative root of the corresponding quadratic equation, and for the $C_{i\ell }=0$ case, the non-negativity constraint on $y_{\lambda }^{\prime }$ yields an update,

$$y_{\lambda ,i\ell }=\text{max}\left(b_{i\ell }/\sigma_{\lambda },0\right).$$

Both the linear and quadratic cases can be merged into the following update equation for $y_{\lambda ,i\ell }$,

(32) $$y_{\lambda ,i\ell }=\left(b_{i\ell }+\sqrt{b_{i\ell }^{2}+4\sigma_{\lambda }C_{i\ell }}\right)/\left(2\sigma_{\lambda }\right)\text{.}$$

Solving the minimization over $y_{\mu }^{\prime }$ in Eq. (31) is more involved because setting the gradient of the objective function to zero results in a transcendental equation, which requires the use of a numerical solver. The objective function is convex in $y_{\mu }^{\prime }$ and its derivatives are easily computed analytically. Thus Newton’s algorithm can be applied to obtain an efficient and accurate solution to Eq. (31). Both the first and second derivatives of the objective function are needed for Newton’s algorithm. Defining ψ to be the objective function of Eq. (31)

$$\psi \left(y_{\mu }^{\prime }\right)=\sum_{i\ell }\left(\text{exp}\left(-y_{\mu ,\ell }^{\prime }\right)\cdot y_{\lambda ,i\ell }+C_{i\ell }\cdot y_{\mu ,\ell }^{\prime }\right)-u_{\mu }^{⊤}y_{\mu }^{\prime }+\frac{\sigma_{\mu }}{2}{\left\|y_{\mu }^{\prime }-P\mu \right\|}^{2},$$

the first derivative of ψ is

(33) $$\frac{\partial \psi \left(y_{\mu }^{\prime }\right)}{\partial y_{\mu ,\ell }^{\prime }}=-\text{exp}\left(-y_{\mu ,\ell }^{\prime }\right)\cdot y_{\lambda ,\ell }+C_{\ell }-u_{\mu }+\sigma_{\mu }\left(y_{\mu ,\ell }^{\prime }-P_{\ell }^{⊤}\mu \right),$$

where

$$y_{\lambda ,\ell }=\underset{i}{\sum }y_{\lambda ,i\ell },\;C_{\ell }=\underset{i}{\sum }C_{i\ell }.$$

The second derivative of ψ is

(34) $$\frac{\partial^{2}\psi \left(y_{\mu }^{\prime }\right)}{\partial y_{\mu ,\ell }^{\prime }{\;}^{2}}=\text{exp}\left(-y_{\mu ,\ell }^{\prime }\right)\cdot y_{\lambda ,\ell }+\sigma_{\mu },$$

which is strictly positive. Thus Newton’s algorithm can be applied without any difficulties with the following update equation

(35) $$y_{\mu ,\ell }^{\prime }\leftarrow y_{\mu ,\ell }^{\prime }-\frac{\partial \psi \left(y_{\mu }^{\prime }\right)}{\partial y_{\mu ,\ell }^{\prime }}{\left(\frac{\partial^{2}\psi \left(y_{\mu }^{\prime }\right)}{\partial y_{\mu ,\ell }^{\prime }{\;}^{2}}\right)}^{-1}.$$

There is also the non-negativity constraint in Eq. (31), and this can be accounted for by thresholding negative values of $y_{\mu ,\ell }^{\prime }$ to zero after the Newton iteration is completed.

The proposed y-update involves two additional levels of iteration. The first additional level of iteration involves alternating between solving Eqs. (30) and (31). In the second additional level of iteration Eq. (31) is solved with the Newton iteration in Eq. (35). Nevertheless, these additional nested iterations do not negatively impact the efficiency of the overall algorithm because all of the iterations for the y-update separate over the components of y. The complete y-update computation takes less effort than computing Tλ, the TOF data of an estimate of the activity map, λ. This is one of the useful aspects of the powerful splitting technique that ADMM exploits.


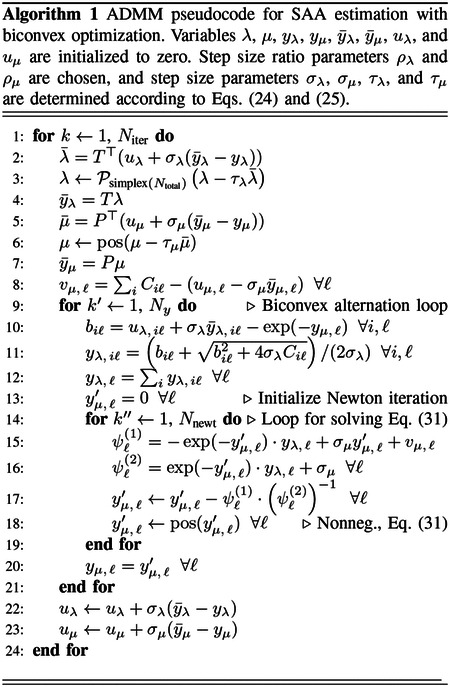


#### ADMM pseudocode for SAA estimation:

The x- ,y-, and u-update equations are assembled into a complete pseudocode given in Algorithm 1. The expensive projection and back-projection computations are collected in as few lines as possible, and their results stored, to avoid unnecessary repetition of these burdensome operations. The simplex projection at line 3 is the optimization problem defined in Eq. (29); efficient computer code for implementing this projection is available from Duchi *et al*. [28]. The first derivative computation from Eq. (33) is performed at lines 8 and 15, where line 8 collects all terms that are not dependent on $y_{\lambda }$ or $y_{\mu }^{\prime }$. The function pos(⋅) in lines 6 and 18 returns the argument if it is nonnegative, otherwise it returns zero. For the results presented in this work, we only consider zero initialization for all of the algorithm variables. The choice of step size ratios $\rho_{\lambda }$ and $\rho_{\mu }$ will impact the convergence rate of the algorithm, and these parameters must be tuned for optimal performance.

### G. ADMM for TV-constrained SAA in TOF-PET

The proposed ADMM framework for solving SAA estimation in TOF-PET allows for great flexibility in imposing convex constraints in the imaging optimization problem. Accordingly, we augment the total annihilation count and nonnegativity constraints in Eq. (21) with additional total variation constraints on the activity and attenuation maps

(36) $$\lambda ,\mu =\underset{\lambda ,\mu }{\text{arg min}}\left\{l(\lambda ,\mu )|{\left\|\lambda \right\|}_{\text{TV}}\leq \gamma_{\lambda },\;{\left\|\mu \right\|}_{\text{TV}}\leq \gamma_{\mu },1^{⊤}\lambda =N_{\text{total}},\;\lambda \geq 0,\;\mu \geq 0\right\},$$

where $∥\cdot {∥}_{\text{TV}}$ is the isotropic TV seminorm; $\gamma_{\lambda }$ and $\gamma_{\mu }$ are the TV constraint values for the activity and attenuation maps, respectively. The additional TV constraints exploit gradient sparsity in in both the activity and attenuation that potentially improves accurate estimation of their corresponding images.

Because the novel aspect of this work is the treatment of the biconvex log-likelihood term, which is explained in detail in Sec. II-F, the ADMM instance for this optimization problem is covered in the Appendix. The ADMM algorithm for TV-constrained SAA estimation (ADMM-TVSAA) is also designed so that it makes use of the same step size ratio parameters as discussed for Algorithm 1. Because of the additional constraints, the TV constraint values $\gamma_{\lambda }$ and $\gamma_{\mu }$ become additional parameters of the algorithm.

### H. Step size scaling of ADMM for SAA

When tuning the step size parameters $\rho_{\lambda }$ and $\rho_{\mu }$ in ADMM-SAA or ADMM-TVSAA, it is important to account for the fact that the the optimal settings for maximum algorithm efficiency change with scaling of the coincidence data C. A practical consequence is that optimally efficient ρ-values would depend on, for example, collection time of the TOF-PET system. In the following results presented in Sec. III, this issue is addressed by normalizing the data C with the factor size $\left(C\right)/∥C{∥}_{2}$. The choice of data normalization is arbitrary because of the following scaling relationship. If we replace the coincidence data C by aC, the same ADMM iterates, up to a scaling, can be obtained by adjusting the constraint and step size parameters as follows:

$$N_{\text{total}}\to aN_{\text{total}},\;\gamma_{\lambda }\to a\gamma_{\lambda },\;\sigma_{\lambda }\to \sigma_{\lambda }/a,\;\tau_{\lambda }\to a\tau_{\lambda },\;\sigma_{\mu }\to a\sigma_{\mu },\;\tau_{\mu }\to \tau_{\mu }/a.$$

With this scaling of the algorithm parameters, the algorithm variables transform as follows:

$$\lambda \to a\lambda ,\;y_{\lambda }\to ay_{\lambda },\;u_{\lambda }\to u_{\lambda },\;\mu \to \mu ,\;y_{\mu }\to y_{\mu },\;u_{\mu }\to au_{\mu }.$$

The scaling transformations can be verified by making the appropriate substitutions into Eqs. (18), (26), (27), (30), and (31), or into the update equations of Algorithm 1.

### I. Huber-penalized MLAA

Use of ADMM allows for nonsmooth terms in the optimization such as use of the TV-norms and complex constraints on the activity and attenuation. The closest comparison from the literature involves a smooth objective function with Huber penalties on the activity and attenuation. Accordingly comparison results are obtained with the penalized-MLAA (P-MLAA) algorithm presented in Mehranian *et al*. [24]. The P-MLAA algorithm implemented here addresses the following optimization problem Huber penalties

(37) $$\lambda ,\mu =\underset{\lambda ,\mu }{\text{arg min}}\left\{l\left(\lambda ,\mu \right)+\beta H_{\delta }\left(\lambda \right)+\gamma H_{\delta }\left(\mu \right)|1^{⊤}\lambda =N_{\text{total}},\;\lambda \geq 0,\;\mu \geq 0\right\},$$

where $H_{\delta }\left(\cdot \right)$ is the Huber penalty with smoothing parameter δ, see for example Nuyts *et al*. [6] for the definition of this penalty function. The P-MLAA algorithm replaces Eq. (5) with the “one step late” update equation developed by Green [30] and introduces a Gibbs prior into Eq. (7) in the manner developed in Nuyts *et al*. [6].

## III. Results with a 2D TOF-PET simulation

**Fig. 1.**
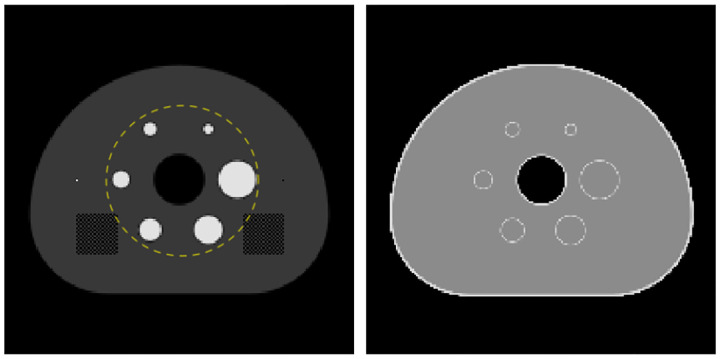
(left) Slice number 40 from the University of Washington Digital Reference Object: activity image in arbitrary units, and (right) attenuation map displayed in the gray scale window $\left[0.075,0.115\right]\;{\text{cm}}^{-1}$. The dashed circle in the activity image indicates the activity distribution used for the investigation of SAA with interior data.

The results demonstrating the ADMM-SAA algorithm are all derived from a 2D simulation using the digital reference object (DRO) shown in Fig. 1 [31]. This digital phantom is cropped to 176×176 image array with physical dimension 30×30 cm^2^. The LORs are arranged in a 2D parallel-beam geometry with 176 views covering a π radian arc, and 176 parallel rays being measured per view with a spacing of 0.17 cm (30/176 ≈ 0.17). The TOF FWHM is taken to be 4.5 cm, which corresponds to a timing resolution of approximately 300 picoseconds. The spacing between TOF window samples is 2.25 cm, and a total of 17 TOF samples are taken per LOR. For the image reconstruction, both the attenuation and activity images are represented on a 176×176 grid. The purpose of the presented results is to demonstrate usage of the ADMM-SAA algorithm and the impact of the TV constraints on the reconstruction of the activity and attenuation.

For the following results, the biconvex alternation loop at line 9 of Algorithm 1 is run for $N_{y}=100$ iterations, and the Newton solver at line 14 is run for $N_{\text{newt}}=10$ iterations. With both of these loop settings, Eq. (23) is solved accurately in a numerical sense. Even with the inner loops being executed with such high iteration numbers, the efficiency of the whole biconvex alternation loop is still high, because all of the computations separate across the vector components. The computational effort for the biconvex alternation loop is $O\left(N_{y}\cdot N_{\text{TOF}}\cdot N_{\text{views}}\cdot \sqrt{N_{\text{pix}}}\right)$ (the Newton loop does not increase the order of this loop because it involves the attenuation sinogram only), and by comparison, computing TOF projection, Tλ, is $O\left(N_{\text{TOF}}\cdot N_{\text{views}}\cdot N_{\text{pix}}\right)$, where $N_{\text{pix }}$ is the total number of pixels and $N_{\text{views}}$ is the number of projection angles. For the small 176×176 images of this study the biconvex loop is of the same order as TOF projection because $N_{y}\approx \sqrt{N_{\text{pix}}}$, but as the data and image size increase, TOF projection becomes the more burdensome computation. It is also possible, in practice, to reduce $N_{y}$ and $N_{\text{newt}}$ and work with inexact solution of Eq. (23) but we do not investigate this option in this work.

### A. SAA from noiseless data

Image reconstruction is performed on noiseless data using the mean counts as the measured data, and ADMM-TVSAA is employed to study the effectiveness for solving the associated inverse problem for two situations: (1) the full activity distribution from the DRO phantom is used as the test object, and (2) the activity distribution is truncated at the yellow, dashed circle in Fig. 1. The second case is a more challenging inverse problem for SAA because recovery of the full attenuation map is complicated by the fact that only LORs that intersect the non-zero activity provide useful data, and accordingly recovery of the attenuation map has a similar degree of difficulty as the interior problem in tomography.

**Fig. 2.**
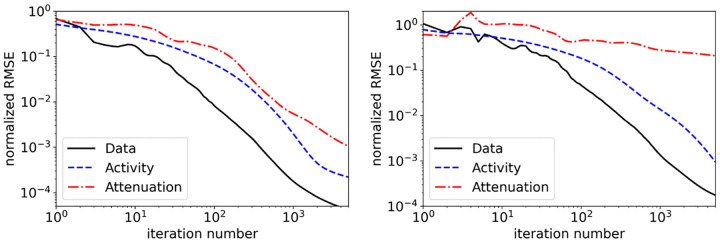
Convergence of ADMM-TVSAA with noiseless TOF-PET data for the case of the full activity distribution (left) and the interior activity distribution (right). The data RMSE is normalized to the mean value of the TOF-PET data, and the activity/attenuation RMSEs are normalized to the mean values of their respective images.

The convergence results for 5000 iterations of ADMM-TVSAA on the phantom with the full activity distribution and the truncated (or interior) activity distribution are shown in Fig. 2. In both cases, the normalized data RMSE and activity RMSE is observed to steadily converge to zero although the activity RMSE is noted to converge more slowly than the data RMSE. Recovering the true attenuation is clearly more challenging; for the full activity distribution case the attenuation RMSE exhibits convergent behavior all the way to the last computed iteration, but for the truncated activity case the global attenuation RMSE does not demonstrate convergent behavior. For truncated activity, however, the attenuation factor derived from the inaccurate attenuation map must have high degree of accuracy since the activity distribution is nicely recovered.

**Fig. 3.**
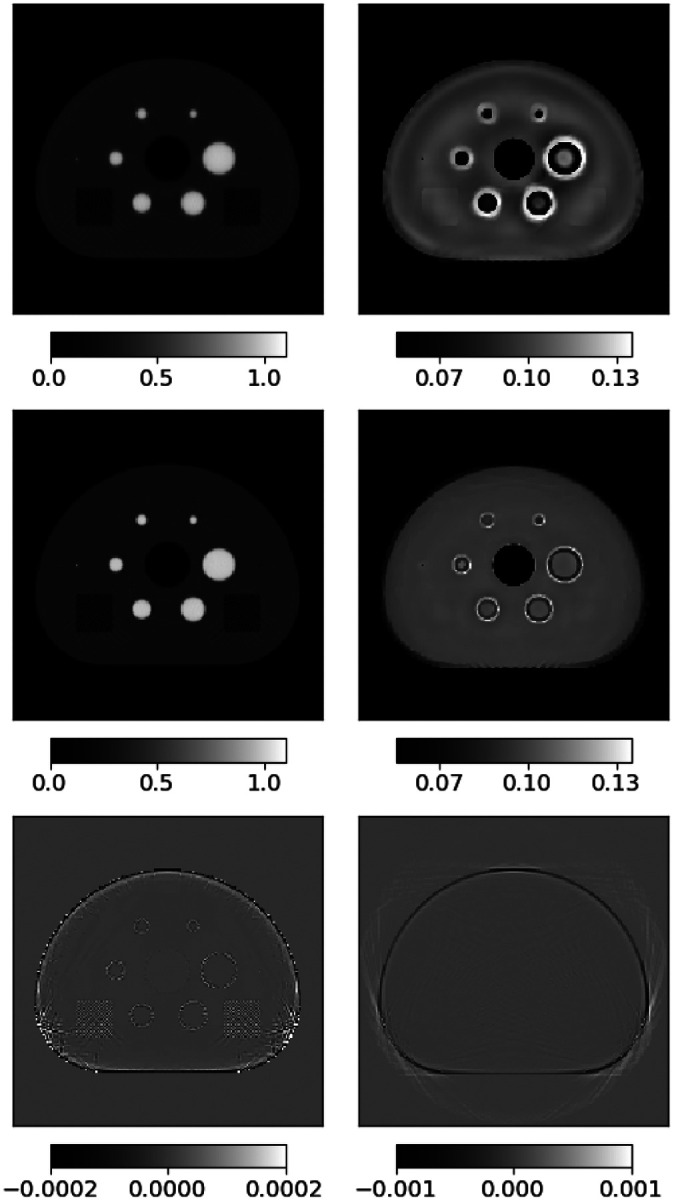
Reconstructed activity (left column) and attenuation (right column) images from noiseless data with ADMM-TVSAA at 50 (top row), 100 (middle row), and 5000 (bottom row) iterations. Because the result at 5000 iterations is visually indistinguishable from the test phantom the difference from the ground truth is displayed in the bottom row. The activity distribution is normalized to 1.0 for the maximum value.

**Fig. 4.**
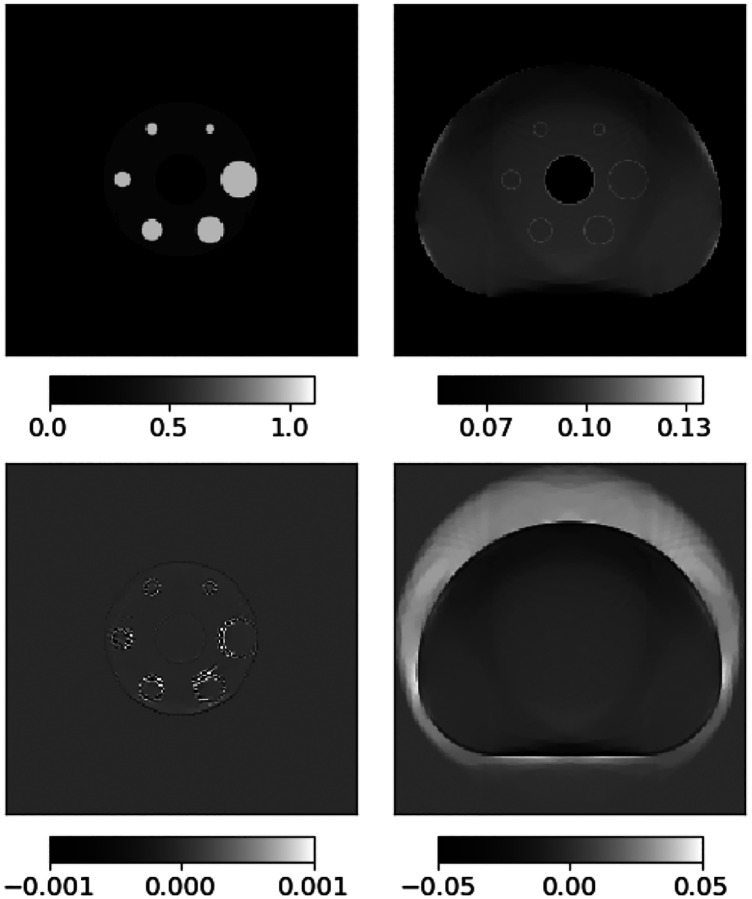
For the results with the interior activity distribution we only show iteration 5000 for the activty (left) and attenuation (right). The actual activity/attenuation images are shown in the top row, and the difference from ground truth is shown in the bottom row.

A series of image estimates are shown for the full activity in Fig. 3 and only the 5000th iteration results are shown for truncated activity in Fig. 4. The difference images at 5000 iterations shows accurate reconstruction of both activity and attenuation for the full distribution case. The attenuation difference in the bottom panels reveals error at the 1% level for the attenuation map near the edges of the phantom. The panels at the earlier iterations provide a sense of the convergence to the solution; specifically the activity images at 50 or 100 iterations closely resemble the result at 5000 iterations. The attenuation map clearly converges more slowly. For the case of truncated activity shown in Fig. 4, the activity image is accurately recovered but it is clear that the attenuation map is not completely recovered. Interestingly, ADMM-TVSAA does seem to be able to recover the support of the attenuation map even if there is substantial error in the outer portions of the image. Moreover, the central portion of the attenuation image in the location where the activity is non-zero does appear to be reconstructed accurately.

### B. SAA from noisy data

The next set of studies focus on SAA with noisy data and only the case of the full activity distribution is considered. Noise realizations are obtained by scaling the mean TOF-PET data so that the total number of measured coincidences is $4\times 10^{6}$; the realization is then obtained by selecting a number of detected coincidences for each time-window sample and LOR, drawn from a Poisson distribution. In demonstrating the use of ADMM-TVSAA, two forms of MLAA provide reference algorithms. The MLAA algorithm described in Sec. II-C is one of the references, where early stopping provides regularization. The P-MLAA algorithm with Huber penalties provides the other reference where the parameters are chosen in such a way that it could conceivably yield similar results as ADMM-TVSAA after 500 iterations. All three algorithms enforce the total annihilation count and non-negativity constraints. The smoothing parameter for P-MLAA’s Huber functions are both chosen to be 0.1% of the phantom maximum value, which is much less than the contrast of structures in either the attenuation or activity maps. In this way the Huber penalties approximate the TV-norm accurately. The penalty parameters are tuned so that the phantom TV values are achieved at 500 iterations of P-MLAA.

**Fig. 5.**
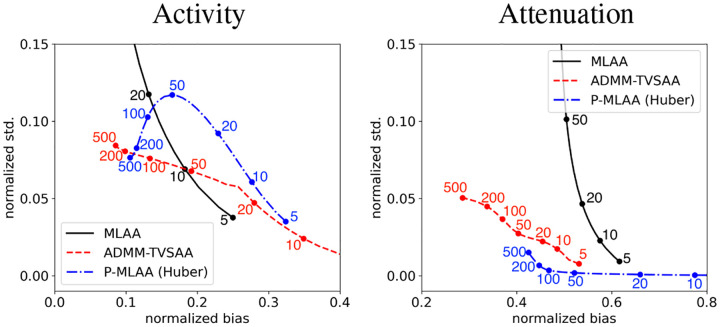
Normalized standard deviation versus normalized bias of the activity (left) and attenuation (right) images as a function of iteration number computed empirically from 100 noise realizations for MLAA, MLAA with Huber penalties, and TV-constrained SAA. Normalization of bias and standard deviation is achieved by dividing by the mean value of the corresponding ground truth image. The labeled dots indicate the iteration numbers for the respective algorithm curves. For TV-constrained ADMM-SAA, curves are shown for activity and attenuation TV constraints set to $\gamma_{\lambda }=1.0$ and $\gamma_{\mu }=1.0$, respectively, where the constraint values are given as a fraction of the ground truth TV values. The Huber penalty parameters for P-MLAA are set so that the resulting activity and attenuation images have nearly the same TV values as the ground truth after 500 iterations.

For a quantitative bias-variance analysis of the activity and attenuation, MLAA, P-MLAA, and ADMM-TVSAA are used to perform SAA on an ensemble of 100 noise realizations of TOF-PET data. The mean and pixel standard deviation are computed and plotted in Fig. 5 as a function of iteration number. The use of the TV-constraints, allows ADMM-TVSAA to achieve activity estimates with low bias and variance as compared with basic maximum-likelihood estimation as implemented with MLAA. Use of explicit Huber penalties with P-MLAA also yields images at 500 iterations that have low bias and variance with respect to MLAA; although the paths in the bias-variance plot for ADMM-TVSAA and P-MLAA are quite different as a function of iteration number. For the particular parameter settings chosen, the ADMM-TVSAA algorithm achieves slightly lower global bias in the activity image with slightly larger variance as compared to P-MLAA. In the activity bias-variance curves, the proximity of the points at 200 and 500 iterations is an indication that the respective ADMM-TVSAA and P-MLAA are near convergence.

The bias-variance curves for the attenuation image reveal a much different behavior than that of the activity image. Both ADMM-TVSAA and P-MLAA substantially improve on the use of MLAA without explicit penalty terms. The variance of the ADMM-TVSAA result is larger than that of P-MLAA, but ADMM-TVSAA achieves a lower bias. It is also clear that 500 iterations is not sufficient to achieve a converged attenuation map because the there is still quite some separation between the points at 200 and 500 iterations for ADMM-TVSAA and P-MLAA. This difference in the rate of convergence of the activity and attenuation images was also seen in the noiseless results shown in Fig. 2. That there can be such a difference in the convergence rate of the two images is due to the fact that the attenuation image only impacts the activity image through the attenuation factor.

**Fig. 6.**
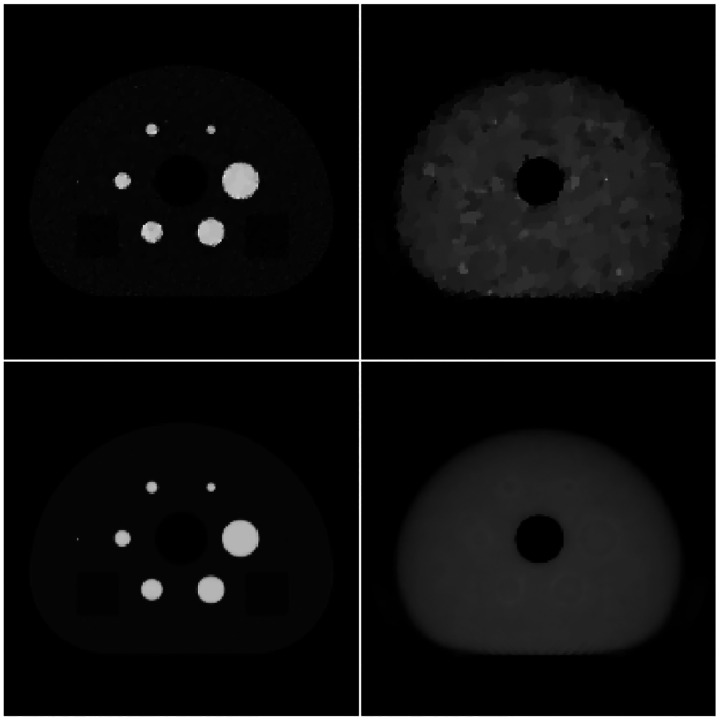
Reconstructed activity (left column) and attenuation (right column) images from noisy data with ADMM-TVSAA. The top row shows a reconstructed set of images from a single noise realization at 500 iterations, and the bottom row shows the corresponding mean over 100 noise realizations. With the activity distribution normalized to 1.0 for the maximum value, the gray scale for the activity images is [0,1.1], and the gray scale for the attenuation is [0.055,0.135].

**Fig. 7.**
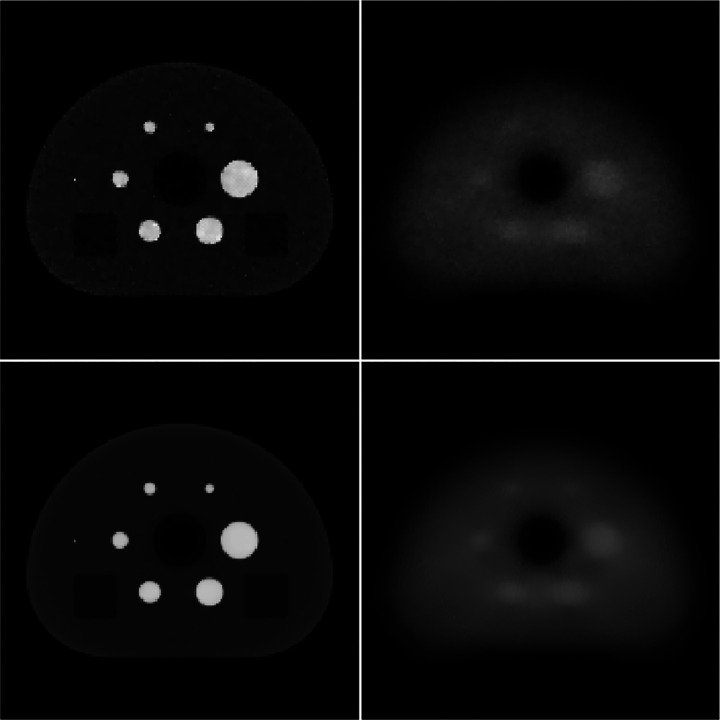
Same as Fig. 6 except that the Huber-regularized MLAA is used to generate the image iterates.

The activity and attenuation images for the present noise studies are shown in Figs. 6 and 7 for ADMM-TVSAA and P-MLAA, respectively. The top row of each figure shows images at 500 iterations for a single noise realization, and the bottom row shows the mean over 100 noise realizations, which reveals the spatial dependence of the image bias. The most notable difference between the two algorithms is in the attenuation images. The ADMM-TVSAA algorithm achieves an attenuation distribution that has the correct support and gray level even if it suffers from noticeable noise artifacts. The result for P-MLAA, however, shows significant bias in the attenuation images and features from the activity distribution clearly bleed through to the attenuation images. The error in the attenuation images, however, may not be critical if the attenuation factors are recovered, and this appears to be the case for both algorithms as the activity images at 500 iterations are accurate. The activity for P-MLAA shows slightly more bias, relative to ADMM-TVSAA, at the phantom border.

**Fig. 8.**
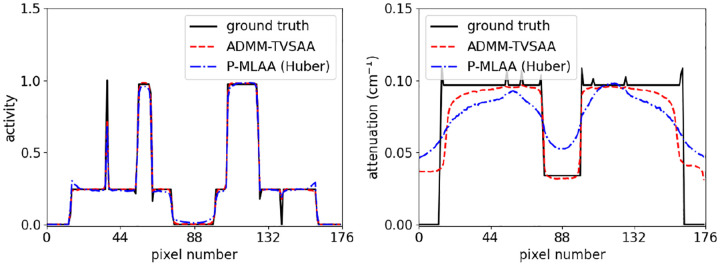
Line profiles through the middle row of pixels comparing the ground truth with the mean reconstructed activity (left) and attenuation (right) images generated at 500 iterations by ADMM-TVSAA and P-MLAA.

The line profiles in Fig. 8 reveal the bias more quantitatively. For ADMM-TVSAA, the mean activity follows the line profile of the ground truth quite closely except for the fact that the sharp features are slightly rounded. The mean attenuation is somewhat accurate except for near the border of the object support where there is significant blurring of the edge. For P-MLAA, the mean activity has a slight increase at the phantom border and a slight filling in of the central cold spot. The attenuation profile for P-MLAA reflects the significant bias that was seen in Fig. 7.

## IV. Discussion

The constrained likelihood model for SAA in TOF-PET enables novel numerical investigation into the SAA inverse problem. In particular, for this work, TV-constraints that exploit gradient sparsity are investigated for simultaneous recovery of activity and attenuation images. From the formulation presented in Sec. II-G, given knowledge of the total annihilation count and TV values of the activity and attenuation, the question is whether or not it is possible to recover the underlying activity and attenuation distributions. For the presented noiseless study where the activity and attenuation have the same support, it appears that both distributions can be accurately recovered. For the case where the activity distribution is interior to the attenuation, the former is recovered accurately while the latter is recovered only within the support of the activity distribution. The study with data noise provides some sense of the stability of the SAA inverse problem when using the TV-constrained likelihood model.

We point out that carrying out inverse problems studies with the penalized likelihood approach shown in Sec. II-I is much more difficult. Aside from the difference between TV and Huber regularization terms, penalized likelihood needs to be investigated in the limit where the penalty parameters approach zero in order to provide the equivalent solution to constrained likelihood when the data are noiseless.

**Fig. 9.**
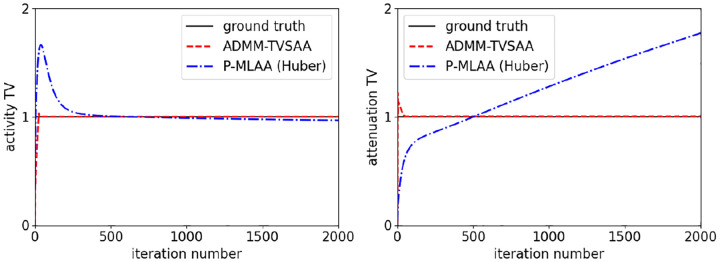
Evolution of activity (left) and attenuation (right) image TV as a function of iteration number for 2000 iterations of ADMM-TVSAA and P-MLAA. The TV values are normalized so that the ground truth phantom values are 1.0.

Even with noisy data, it is difficult to use the penalized-likelihood approach for achieving the same solution as the constrained-likelihood model. This is illustrated in the evolution of the activity and attenuation image TV shown in Fig. 9. These results correspond to the noise study that yielded the single noise realization images in Figs. 6 and 7 except that the iteration number is extended to 2000 . For ADMM-TVSAA, the image TVs reach their constraint values quickly and maintain these values. For P-MLAA, the desired constraint values are achieved at 500 iterations because a two-dimensional penalty parameter search was performed to achieve this goal. Iterating further with P-MLAA causes the image TV to change. This change is not large for the activity because this image is nearly converged, but for the attenuation the change in TV is rather large. In order to come closer to the desired constraint values with P-MLAA, the penalty parameter search would need to be performed at larger iteration numbers, increasing the computational burden.

So far this discussion has focused on algorithms for investigating the TOF-PET SAA inverse problem, which can be quite different than their use for image reconstruction in real data scenarios. For an actual scan, ground truth is not available and mathematical accuracy of the solution may not correlate well with the imaging task of interest. Parameters of ADMM-TVSAA or P-MLAA need to be optimized on a metric reflecting performance on this imaging task. Also, because accurate solution is not necessarily the goal, early stopping is almost always used in practical applications of iterative image reconstruction. In this sense, iteration number becomes a parameter to tune and the practical difference between these two algorithms is the different paths they take in approaching their respective solutions. We also point out that we have shown results for P-MLAA using Green’s one step late algorithm, and use of other solvers such as ADMM with SQS [25] may lead to improved performance of P-MLAA.

The presented study made use of a relatively simple test object that may favor the use of TV constraints. For realistic activity distributions, such constraints can still be employed effectively as is shown in Refs. [15] and [16], where human PET data is reconstructed using such constraints.

## V. Conclusion

In this work, an ADMM framework is developed that can be applied to nonsmooth and nonconvex optimization problems that arise in imaging. The particular form of nonconvexity addressed is when the optimization problem has a biconvex structure. The imaging problem posed by simultaneous estimation of the activity and attenuation (SAA) in time-of-flight positron emission tomography (TOF-PET) has such a structure. Using this ADMM framework, a limited study on the impact of total variation (TV) constraints on the activity and attenuation for the SAA problem is presented. The use of both of these constraints is seen to help stabilize the SAA inverse problem. The shown results are intended to demonstrate the ADMM-TVSAA algorithm and its potential in solving the SAA inverse problem with the use of TV constraints on the activity and attenuation. While we have shown results only for ADMM-TVSAA, the ADMM framework is easily extended to include other nonsmooth, convex terms. Further study varying the test phantom and TOF-PET setup are needed to obtain a more comprehensive picture of the SAA inverse problem.

## Data Availability

The implementation of the algorithms, which are presented in this article, and the code, which generates the figures, are available at: https://github.com/zhimeir/saa_admm_paper.

## Appendix

### ADMM for TVSAA in TOF-PET

In this Appendix, the details for the instantiation of ADMM for TV-constraineed SAA estimation (ADMM-TVSAA) is explained. The optimization problem of interest is Eq. (36) from the main text, which we restate here

(A1)
$$\lambda ,\mu =\underset{\lambda ,\mu }{\text{arg min}}\left\{l\left(\lambda ,\mu \right)|{\left\|D\lambda \right\|}_{1}\leq \gamma_{\lambda },\;{\left\|D\mu \right\|}_{1}\leq \gamma_{\mu }1^{⊤}\lambda =N_{\text{total}},\;\lambda \geq 0,\;\mu \geq 0\right\},$$

where D is the discretization of the spatial gradient operator, and ${\left\|Dx\right\|}_{1}$ is the anisotropic TV of x. To map the optimization problem onto the generic ADMM optimization in Eq. (14), the primal, splitting, and dual variables x,y, and u, are respectively assigned as

$$x=\left(\begin{array}{c} \lambda \\ \mu \end{array}\right),\;y=\left(\begin{array}{c} y_{\lambda }\\ z_{\lambda }\\ y_{\mu }\\ z_{\mu } \end{array}\right),\;u=\left(\begin{array}{c} u_{\lambda }\\ v_{\lambda }\\ u_{\mu }\\ v_{\mu } \end{array}\right),$$

The linear system A is assigned as

$$A=\left(\begin{array}{cc} T & 0\\ \nu_{\lambda }D & 0\\ 0 & P\\ 0 & \nu_{\mu }D \end{array}\right),$$

where

(A2)
$$\nu_{\lambda }={\left\|T\right\|}_{2}/{\left\|D\right\|}_{2},\;\nu_{\mu }={\left\|P\right\|}_{2}/{\left\|D\right\|}_{2},$$

are constants that normalize the gradient matrices to the projection matrices.

As with the SAA problem, the convex function f is used to represent the constraint on the total number of annihilations by setting

$$f\left(\lambda ,\mu \right)=\delta \left(1^{⊤}\lambda =N_{\text{total}}\right)+\delta \left(\lambda \geq 0\right)+\delta \left(\mu \geq 0\right).$$

The biconvex function g accounts for the remaining terms in Eq. (A1)

(A3)
$$g\left(y_{\lambda },y_{\mu }\right)=L\left(y_{\lambda },y_{\mu }\right)+\delta \left({\left\|z_{\lambda }\right\|}_{1}\leq \nu_{\lambda }\gamma_{\lambda }\right)+\delta \left({\left\|z_{\mu }\right\|}_{1}\leq \nu_{\mu }\gamma_{\mu }\right),$$

where the biconvex function $L\left(y_{\lambda },y_{\mu }\right)$ is defined in Eq. (23) and the TV constraint values have also been scaled to reflect the normalization of D.

#### Parametrization of the step-sizes:

With the modified system matrix A, the metric from Eq. (14) becomes

$$H_{f}=\left(\begin{array}{cc} H_{\lambda } & 0\\ 0 & H_{\mu } \end{array}\right),\;H_{\lambda }=\frac{I}{\tau_{\lambda }}-\sigma_{\lambda }\left(T^{⊤}T+\nu_{\lambda }^{2}D^{⊤}D\right),\;H_{\mu }=\frac{I}{\tau_{\mu }}-\sigma_{\mu }\left(P^{⊤}P+\nu_{\mu }^{2}D^{⊤}D\right),$$

and the step-size parameters are chosen so that

$$\sigma_{\lambda }\tau_{\lambda }={\left({\left\|T\right\|}_{2}^{2}+\nu_{\lambda }^{2}{\left\|D\right\|}_{2}^{2}\right)}^{-1},\;\sigma_{\mu }\tau_{\mu }={\left({\left\|P\right\|}_{2}^{2}+\nu_{\mu }^{2}{\left\|D\right\|}_{2}^{2}\right)}^{-1}.$$

As with ADMM-SAA the step size ratios, $\rho_{\lambda }$ and $\rho_{\mu }$, need to be determined by a similar grid search to what is shown in Sec. IIIA. The grid search performed for ADMM-SAA provides a good initial starting point for the step-size parameter search for ADMM-TVSAA when the parameters $\nu_{\lambda }$ and $\nu_{\mu }$ are determined by Eq. (A2). Accordingly, the step size parameters for ADMM-TVSAA are

$$\sigma_{\lambda }=\rho_{\lambda }{\left({\left\|T\right\|}_{2}^{2}+\nu_{\lambda }^{2}{\left\|D\right\|}_{2}^{2}\right)}^{-1},\;\tau_{\lambda }=\rho_{\lambda }^{-1}{\left({\left\|T\right\|}_{2}^{2}+\nu_{\lambda }^{2}{\left\|D\right\|}_{2}^{2}\right)}^{-1}\;\sigma_{\mu }=\rho_{\mu }{\left({\left\|P\right\|}_{2}^{2}+\nu_{\mu }^{2}{\left\|D\right\|}_{2}^{2}\right)}^{-1},\;\tau_{\mu }=\rho_{\mu }^{-1}{\left({\left\|P\right\|}_{2}^{2}+\nu_{\mu }^{2}{\left\|D\right\|}_{2}^{2}\right)}^{-1}.$$

#### The y-update:

The g function in Eq. (A3) separates into a biconvex function in $y_{\lambda }$ and $y_{\mu }$, and convex functions in $z_{\lambda }$ and $z_{\mu }$. The biconvex terms are treated in exactly the same way as the ADMM-SAA presentation in Sec. IIF. Focusing on the last two convex terms in Eq. (A3) yields the optimization problems

(A4)
$$z_{\lambda }=\underset{z_{\lambda }^{\prime }}{\text{arg min}}\left\{\delta \left({\left\|z_{\lambda }^{\prime }\right\|}_{1}\leq \nu_{\lambda }\gamma_{\lambda }\right)-v_{\lambda }^{⊤}z_{\lambda }^{\prime }+\frac{\sigma_{\lambda }}{2}{\left\|z_{\lambda }^{\prime }-\nu_{\lambda }D\lambda \right\|}^{2}\right\},$$

and

(A5)
$$z_{\mu }=\underset{z_{\mu }^{\prime }}{\text{arg min}}\left\{\delta \left({\left\|z_{\mu }^{\prime }\right\|}_{1}\leq \nu_{\mu }\gamma_{\mu }\right)-v_{\mu }^{⊤}z_{\mu }^{\prime }+\frac{\sigma_{\mu }}{2}{\left\|z_{\mu }^{\prime }-\nu_{\mu }D\mu \right\|}^{2}\right\}.$$

Because both of these problems are identical, we focus on Eq. (A4). The objective function consists of a quadratic function and an indicator function that enforces the $\ell_{1}$ constraint on $z_{\lambda }^{\prime }$. Furthermore, the quadratic function has uniform curvature, i.e. a Hessian matrix that is proportional to the identity matrix. For this special case, the solution to Eq. (A4) is obtained in a two-step process that involves finding the minimizer of the unconstrained quadratic function, then projecting the result to the closest $z_{\lambda }^{\prime }$ that satisfies the $\ell_{1}$ constraint. The unconstrained minimizer is given by

$$z_{\lambda }^{\prime \prime }=v_{\lambda }/\sigma_{\lambda }+\nu_{\lambda }D\lambda$$

and the solution to Eq. (A4) becomes

$$z_{\lambda }^{\prime }={𝒫}_{L1\left(\nu_{\lambda }\gamma_{\lambda }\right)}\left(z_{\lambda }^{\prime \prime }\right),\;L1\left(r\right)=\left\{z|{\left\|z\right\|}_{1}\leq r\right\},$$

where ${𝒫}_{L1\left(\nu_{\lambda }\gamma_{\lambda }\right)}\left(\cdot \right)$ denotes projection onto the $\ell_{1}$-ball of “radius” $\nu_{\lambda }\gamma_{\lambda }$. An efficient algorithm for performing this projection is presented in Ref. [28], or it can also be accomplished by vector shrinkage and use of a root finding algorithm to determine the shrinkage parameter to attain an $\ell_{1}$-norm of $\nu_{\lambda }\gamma_{\lambda }$. The update equations for $z_{\lambda }$ and $z_{\mu }$ are

$$z_{\lambda }\leftarrow {𝒫}_{L1\left(\nu_{\lambda }\gamma_{\lambda }\right)}\left(v_{\lambda }/\sigma_{\lambda }+\nu_{\lambda }D\lambda \right),\;z_{\mu }\leftarrow {𝒫}_{L1\left(\nu_{\mu }\gamma_{\mu }\right)}\left(v_{\mu }/\sigma_{\mu }+\nu_{\mu }D\mu \right).$$

#### ADMM pseudocode for TV-constrained SAA estimation:

The derivations in this appendix yield the additional steps that are needed to formulate the pseudocode shown in Algorithm 2 from the ADMM-SAA pseudocode in Algorithm 1.
