# Supplementary material for: Simultaneous activity and attenuation estimation in TOF-PET with TV-constrained nonconvex optimization
Source: ArXiv. 2023 Mar 29:arXiv:2303.17042v1. Preprint. [Version 1] (PMC10081343)
Supplement: Supplementary file 1 [file NIHPP2303.17042v1-supplement-1.pdf]

Simultaneous activity and attenuation estimation in TOF-PET with TV-constrained nonconvex optimization: Supplemental Material

ADMM for TVSAA in TOF-PET

In this Supplement, the details for the instantiation of ADMM for TV-constrained SAA estimation (ADMM-TVSAA) are explained. The optimization problem of interest is Eq. (39) from the main text, which we restate here

$$\lambda, \mu = \arg\min_{\lambda,\mu} \Big\{ l(\lambda,\mu) \;|\; \|D\lambda\|_1 \le \gamma_\lambda,\;\; \|D\mu\|_1 \le \gamma_\mu, \;\; \mathbf{1}^\top \lambda = N_{\text{total}},\;\; P\mu \ge 0 \Big\}, \quad \text{(S1)}$$

where D is the discretization of the spatial gradient operator, and $\|Dx\|_1$ is the anisotropic TV of x. To map the optimization problem onto the generic ADMM optimization in Eq. (14), the primal, splitting, and dual variables x, y, and u, are respectively assigned as

$$x = \begin{pmatrix} \lambda \\ \mu \end{pmatrix}, \quad y = \begin{pmatrix} y_\lambda \\ z_\lambda \\ y_\mu \\ z_\mu \end{pmatrix}, \quad u = \begin{pmatrix} u_\lambda \\ v_\lambda \\ u_\mu \\ v_\mu \end{pmatrix},$$

The linear system A is assigned as

$$A = \begin{pmatrix} T & 0 \\ \nu_\lambda D & 0 \\ 0 & P \\ 0 & \nu_\mu D \end{pmatrix},$$

where

$$\nu_\lambda = \|T\|_2 / \|D\|_2, \quad \nu_\mu = \|P\|_2/\|D\|_2, \quad \text{(S2)}$$

are constants that normalize the gradient matrices to the projection matrices.

As with the SAA problem, the convex function f is used to represent the constraint on the total number of annihilations by setting

$$f(\lambda,\mu) = \delta(\mathbf{1}^\top \lambda = N_{\text{total}}).$$

The biconvex function g accounts for the remaining terms in Eq. (S1)

$$g(y_\lambda, y_\mu) = L(y_\lambda, y_\mu) + \delta(y_\mu \ge 0) + \delta(\|z_\lambda\|_1 \le \nu_\lambda\gamma_\lambda) + \delta(\|z_\mu\|_1 \le \nu_\mu \gamma_\mu), \quad \text{(S3)}$$

where the biconvex function $L(y_\lambda, y_\mu)$ is defined in Eq. (2) and the TV constraint values have also been scaled to reflect the normalization of D.

Parametrization of the step-sizes: With the modified system matrix A, the metric from Eq. (13) becomes

$$H_f = \begin{pmatrix} H_\lambda & 0 \\ 0 & H_\mu \end{pmatrix},$$
$$H_\lambda = \frac{I}{\tau_\lambda} - \sigma_\lambda (T^\top T + \nu_\lambda^2 D^\top D),$$
$$H_\mu = \frac{I}{\tau_\mu} - \sigma_\mu (P^\top P + \nu_\mu^2 D^\top D),$$

and the step-size parameters are chosen so that

$$\sigma_\lambda \tau_\lambda = (\|T\|_2^2 + \nu_\lambda^2 \|D\|_2^2)^{-1}, \quad \sigma_\mu\tau_\mu = (\|P\|_2^2 + \nu_\mu^2\|D\|_2^2)^{-1}.$$

We have found empirically that the step size ratios, $\rho_\lambda = 0.01$ and $\rho_\mu = 100$, determined for ADMM-SAA in Sec. IIIA provide sufficient efficiency for ADMM-TVSAA when the parameters ν_λ and ν_μ are determined by Eq. (S2). Accordingly, the step size parameters for ADMM-TVSAA are

$$\sigma_\lambda = \rho_\lambda (\|T\|_2^2 + \nu_\lambda^2\|D\|_2^2)^{-1},$$
$$\tau_\lambda = \rho_\lambda^{-1} (\|T\|_2^2 + \nu_\lambda^2\|D\|_2^2)^{-1},$$
$$\sigma_\mu = \rho_\mu (\|P\|_2^2 + \nu_\mu^2\|D\|_2^2)^{-1},$$
$$\tau_\mu = \rho_\mu^{-1} (\|P\|_2^2 + \nu_\mu^2\|D\|_2^2)^{-1}.$$

The x-update: For the TVSAA problem in TOF-PET the instantiation of the x-update in Eq. (15) is a straightforward generalization of the presentation in Sec. IIF, and we only state the final update equations. The μ-update of ADMM-SAA in Eq. (28) becomes

$$\mu \leftarrow \mu - \tau_\mu (P^\top \bar{u}_\mu + \nu_\mu D^\top \bar{v}_\mu), \quad \text{(S4)}$$
$$\bar{u}_\mu = u_\mu + \sigma_\mu (P\mu - y_\mu),$$
$$\bar{v}_\mu = v_\mu + \sigma_\mu(\nu_\mu D\mu - z_\mu),$$

for ADMM-TVSAA. Likewise, the λ-update of ADMM-SAA in Eq. (30) becomes

$$\lambda \leftarrow \lambda - \tau_\lambda (\nu \mathbf{1} + T^\top \bar{u}_\lambda + \nu_\lambda D^\top \bar{v}_\lambda), \quad \text{(S5)}$$
$$\nu = \frac{1}{\tau_\lambda N_{\text{pix}}} \left(\mathbf{1}^\top\lambda - N_{\text{total}} - \tau_\lambda \mathbf{1}^\top (T^\top \bar{u}_\lambda + \nu_\lambda D^\top \bar{v}_\lambda)\right).$$

The y-update: The g function in Eq. (S3) separates into a biconvex function in y_λ and y_μ, and convex functions in z_λ and z_μ. The biconvex terms are treated in exactly the same way as the ADMM-SAA presentation in Sec. IIF. Focusing on the last two convex terms in Eq. (S3) yields the optimization problems

$$z_\lambda = \arg\min_{z'_\lambda} \Big\{ \delta(\|z'_\lambda\|_1 \le \nu_\lambda\gamma_\lambda) - v_\lambda^\top z'_\lambda + \frac{\sigma_\lambda}{2} \|z'_\lambda - \nu_\lambda D\lambda\|^2 \Big\}, \quad \text{(S6)}$$

and

$$z_\mu = \arg\min_{z'_\mu} \Big\{ \delta(\|z'_\mu\|_1 \le \nu_\mu\gamma_\mu) - v_\mu^\top z'_\mu + \frac{\sigma_\mu}{2} \|z'_\mu - \nu_\mu D\mu\|^2 \Big\}. \quad \text{(S7)}$$

Because both of these problems are identical, we focus on Eq. (S6). The objective function consists of a quadratic function and an indicator function that enforces the ℓ_1 constraint on z'_λ. Furthermore, the quadratic function has uniform curvature, i.e. a Hessian matrix that is proportional to the identity matrix. For this special case, the solution to Eq. (S6) is obtained in a two-step process that involves finding the minimizer of the unconstrained quadratic function, then projecting the result to the closest z'_λ that satisfies the ℓ_1 constraint. The unconstrained minimizer is given by

$$z''_\lambda = v_\lambda / \sigma_\lambda + \nu_\lambda D\lambda,$$

and the solution to Eq. (S6) becomes

$$z'_\lambda = \mathcal{P}_{L1(\nu_\lambda\gamma_\lambda)}(z''_\lambda), \quad L1(r) = \{z \mid \|z\|_1 \le r\},$$

where $\mathcal{P}_{L1(\nu_\lambda \gamma_\lambda)}(\cdot)$ denotes projection onto the ℓ_1-ball of "radius" $\nu_\lambda \gamma_\lambda$. An efficient algorithm for performing this projection is presented in Ref. [1], or it can also be accomplished by vector shrinkage and use of a root finding algorithm to determine the shrinkage parameter to attain an ℓ_1-norm of $\nu_\lambda \gamma_\lambda$. The update equations for z_λ and z_μ are

$$z_\lambda \leftarrow \mathcal{P}_{L1(\nu_\lambda \gamma_\lambda)}(v_\lambda / \sigma_\lambda + \nu_\lambda D\lambda),$$
$$z_\mu \leftarrow \mathcal{P}_{L1(\nu_\mu \gamma_\mu)}(v_\mu / \sigma_\mu + \nu_\mu D\mu).$$

The u*-update:* For ADMM-TVSAA, Eq. (17) becomes

$$u_\lambda \leftarrow u_\lambda + \sigma_\lambda (T\lambda - y_\lambda),$$
$$u_\mu \leftarrow u_\mu + \sigma_\mu (P\mu - y_\mu),$$
$$v_\lambda \leftarrow v_\lambda + \sigma_\lambda (\nu_\lambda D\lambda - z_\lambda),$$
$$v_\mu \leftarrow v_\mu + \sigma_\mu (\nu_\mu D\mu - z_\mu).$$

Algorithm 2 ADMM pseudocode for TV-constrained SAA estimation. Variables λ, μ, y_λ, y_μ, z_λ, z_μ,$\bar{y}_\lambda$, $\bar{y}_\mu$, $\bar{z}_\lambda$, $\bar{z}_\mu$, u_λ, u_μ, v_λ, and v_μ are initialized to zero.

for $k \leftarrow 1,\, N_{\text{iter}}$ **do**
  $\bar{\lambda}_1 = T^\top (u_\lambda + \sigma_\lambda (\bar{y}_\lambda - y_\lambda))$
  $\bar{\lambda}_2 = \nu_\lambda D^\top (v_\lambda + \sigma_\lambda (\bar{z}_\lambda - z_\lambda))$
  $\nu = \frac{1}{\tau_\lambda N_{\text{pix}}} \left(\mathbf{1}^\top \lambda - N_{\text{total}} - \tau_\lambda \mathbf{1}^\top (\bar{\lambda}_1 + \bar{\lambda}_2) \right)$
  $\lambda \leftarrow \lambda - \tau_\lambda (\nu \mathbf{1} + \bar{\lambda}_1 + \bar{\lambda}_2)$
  $\bar{y}_\lambda = T\lambda$
  $\bar{z}_\lambda = \nu_\lambda D\lambda$
  $\bar{\mu}_1 = P^\top (u_\mu + \sigma_\mu (\bar{y}_\mu - y_\mu))$
  $\bar{\mu}_2 = \nu_\mu D^\top (v_\mu + \sigma_\mu (\bar{z}_\mu - z_\mu))$
  $\mu \leftarrow \mu - \tau_\mu (\bar{\mu}_1 + \bar{\mu}_2)$
  $\bar{y}_\mu = P\mu$
  $\bar{z}_\mu = \nu_\mu D\mu$
  $v_{\mu,\ell} = \sum_i C_{i\ell} - (u_{\mu,\ell} - \sigma_\mu \bar{y}_{\mu,\ell}) \;\; \forall \ell$
  for $k' \leftarrow 1,\, N_y$ **do**
    $b_{i\ell} = u_{\lambda,i\ell} + \sigma_\lambda \bar{y}_{\lambda,i\ell} - \exp(-y_{\mu,\ell}) \;\; \forall i, \ell$
    $y_{\lambda,i\ell} = \left(b_{i\ell} + \sqrt{b_{i\ell}^2 + 4\sigma_\lambda C_{i\ell}} \right) / (2\sigma_\lambda) \;\; \forall i, \ell$
    $y_{\lambda,\ell} = \sum_i y_{\lambda,i\ell} \;\; \forall \ell$
    $y'_{\mu,\ell} = 0 \;\; \forall \ell$
    for $k'' \leftarrow 1,\, N_{\text{newt}}$ **do**
      $\psi_\ell^{(1)} = -\exp(-y'_{\mu,\ell}) \cdot y_{\lambda,\ell} + \sigma_\mu y'_{\mu,\ell} + v_{\mu,\ell}$
      $\psi_\ell^{(2)} = -\exp(-y'_{\mu,\ell}) \cdot y_{\lambda,\ell} + \sigma_\mu \;\; \forall \ell$
      $y'_{\mu,\ell} \leftarrow y'_{\mu,\ell} - \psi_\ell^{(1)} \cdot \left(\psi_\ell^{(2)} \right)^{-1} \;\; \forall \ell$
      $y'_{\mu,\ell} \leftarrow \mathrm{pos}(y'_{\mu,\ell}) \;\; \forall \ell$
    end for
    $y_{\mu,\ell} = y'_{\mu,\ell} \;\; \forall \ell$
  end for
  $z_\lambda \leftarrow \mathcal{P}_{L1(\nu_\lambda \gamma_\lambda)}(v_\lambda / \sigma_\lambda + \bar{z}_\lambda)$
  $z_\mu \leftarrow \mathcal{P}_{L1(\nu_\mu \gamma_\mu)}(v_\mu / \sigma_\mu + \bar{z}_\mu)$
  $u_\lambda \leftarrow u_\lambda + \sigma_\lambda (\bar{y}_\lambda - y_\lambda)$
  $u_\mu \leftarrow u_\mu + \sigma_\mu (\bar{y}_\mu - y_\mu)$
  $v_\lambda \leftarrow v_\lambda + \sigma_\lambda (\bar{z}_\lambda - z_\lambda)$
  $v_\mu \leftarrow v_\mu + \sigma_\mu (\bar{z}_\mu - z_\mu)$
end for

ADMM pseudocode for TV-constrained SAA estimation: The derivations in this appendix yield the additional steps that are needed to formulate the pseudocode shown in Algorithm S1 from the ADMM-SAA pseudocode in Algorithm 1. The step size ratio parameters ρ_λ and ρ_μ are determined by performing a small grid search centered on the values obtained for the grid search described in Sec. IIIA. The values used in generating the results for ADMM-TVSAA are as follows: when both λ- and μ-TV constraints are active, $\sigma_\mu = 50$ and $\sigma_\lambda = 0.01$; when only the μ-TV constraint is active, $\sigma_\mu = 100$ and $\sigma_\lambda = 0.01$; and when only the λ-TV constraint is active, $\sigma_\mu = 200$ and $\sigma_\lambda = 0.01$.

References

[1] J. Duchi, S. Shalev-Shwartz, Y. Singer, and T. Chandra, "Efficient projections onto the ℓ_1-ball for learning in high dimensions," *Proc. 25th Int. Conf Mach. Learn.*, pp. 272–279, 2008.
